# Assessment of a New Copper-Based Formulation to Control Esca Disease in Field and Study of Its Impact on the Vine Microbiome, Vine Physiology and Enological Parameters of the Juice

**DOI:** 10.3390/jof8020151

**Published:** 2022-01-31

**Authors:** Vincenzo Mondello, Christelle Lemaître-Guillier, Patricia Trotel-Aziz, Régis Gougeon, Alberto Acedo, Philippe Schmitt-Kopplin, Marielle Adrian, Cátia Pinto, Olivier Fernandez, Florence Fontaine

**Affiliations:** 1 Unité Résistance Induite et Bioprotection des Plantes EA 4707 USC INRAE 1488, Université de Reims Champagne-Ardenne, SFR Condorcet FR CNRS 3417, 51100 Reims, France; patricia.trotel-aziz@univ-reims.fr (P.T.-A.); olivier.fernandez@univ-reims.fr (O.F.); 2 Agroécologie, Institut Agro Dijon, CNRS, Université de Bourgogne, INRAE, Université de Bourgogne Franche-Comté, 21000 Dijon, France; christelle.guillier@inrae.fr (C.L.-G.); marielle.adrian@u-bourgogne.fr (M.A.); 3 Institut Universitaire de la Vigne et du Vin, UMR PAM Université de Bourgogne, Institut Agro Dijon, Rue Claude Ladrey, CEDEX BP 27877, 21078 Dijon, France; regis.gougeon@u-bourgogne.fr; 4 Biome Makers, 890 Embarcadero Drive, West Sacramento, CA 95605, USA; acedo@biomemakers.com; 5 Analytical BioGeoChemistry, Helmholtz Zentrum München, German Research Center for Environmental Health, 85764 Neuherberg, Germany; schmitt-kopplin@helmholtz-muenchen.de; 6 Associação SFCOLAB—Laboratório Colaborativo para a Inovação Digital na Agricultura, Rua Cândido dos Reis nº 1. Espaço SFCOLAB, 2560-312 Torres Vedras, Portugal; catia.pinto@sfcolab.org

**Keywords:** vine, esca incidence, microbiota, metabolome, transcriptome, copper fungicides

## Abstract

Copper-based preparations have been used for more than 100 years in viticulture to control downy mildew caused by *Plasmopara viticola*. LC2017, and a new low-copper-based formulation, has been developed to control grapevine trunk diseases (GTDs). Previous greenhouse studies showed the potential of LC2017 to control GTDs by both fungistatic and plant defense elicitor effects. Here, we further characterize the effects of LC2017 in the field determining its impact on: (i) incidence of Esca, (ii) the vine microbiome, (iii) the vine physiology and (iv) enological parameters of juices. We observed a progressive decrease of cumulate Esca incidence in treated vines over the years with annual fluctuation related to the known erratic emergence of GTD symptoms. Neither harmful effects of LC2017 on the vine microbiota, nor on vine physiology were observed (at both transcriptomic and metabolomic levels). Similarly, no impact of LC2017 was observed on the enological properties of berries except for sugar content in juice from esca-diseased vines. The most important result concerns the transcriptomic profiles: that of diseased and LC2017 treated vines differs from that of disease untreated ones, showing a treatment effect. Moreover, the transcriptomic profile of diseased and LC2017-treated vines is similar to that of untreated asymptomatic vines, suggesting control of the disease.

## 1. Introduction

Copper (Cu) is one of the oldest and the most common active ingredients used in agriculture to control plant diseases. Its use began in 1880s when Millardet discovered the ability of a lime-copper mixture, known as Bordeaux mixture, to control grapevine downy mildew caused by *Plasmopara viticola* [1,2]. As a consequence, the Bordeaux mixture became the first fungicide to be used on a large scale on a worldwide level. Despite the evolution of plant protection products (PPPs), the use of copper-based PPPs remain essential in modern viticulture for several reasons, especially their relatively low cost and fungicidal effect. Indeed, cupric PPPs are always recognized as the most efficient products authorized in organic viticulture to control downy mildew, one of the most relevant and recurrent grapevine diseases [3]. Furthermore, Cu has the advantage of having a multi-site biocide action that does not entail any risk of fungicide-resistant strains, by contrast with unisite fungicides that target specific metabolic functions [4]. Nevertheless, it has the disadvantage of being a contact fungicide and, therefore, leachable due to many treatments over rainy years.

The appearance of undesired environmental issues, related to the toxic effects caused by Cu accumulation in soils and potential consecutive contamination of the aquatic environment [5,6], is forcing reconsideration of copper use in crop protection strategies. The issue is particularly urgent in viticulture where the number of copper-based treatments can vary during a vegetative season representing a maximum amount of 4 kg∙ha^−1^∙year^−1^ of copper distributed in the European Union (1981/2018 EC regulation). It is worth mentioning that in the past the European Union frequently allowed 30 kg∙ha^−1^ per every 5 years [7]. As a consequence, the Cu concentration can reach very high levels in soil (up to 3000 mg Cu∙kg^−1^ of soil [8]) and have negative effects on soil biota [6]. For these reasons, copper is now considered as “candidate for substitution” (art. 24 EC Regulation n. 1107/2009) by the European Community (EC) and its use is now limited (max. 28 kg∙ha^−1^ of metallic copper within 7 years, or 4 kg∙ha^−1^∙year^−1^—1981/2018 EC regulation).

To maintain the use of cupric fungicides and to respect the EU limit in viticulture, many strategies related to PPPs formulation are currently being evaluated such as: (i) Cu micronization, (ii) its progressive release and resistance to rainwater, and (iii) the modulation of Cu bioavailability according to environmental conditions, by combining copper with other substances (e.g., zeolites, clay, terpene alcohol- [9]). To the latter belongs the new copper-based product hydroxyapatite + copper (HA+Cu(II), namely LC2017) described by Battiston et al. [10,11,12]. Copper, in form of sulfate pentahydrate salt (3.5% *v*/*v*) is transported throughout the plant by hydroxyapatite (HA), a carrier molecule, that limits the leachable aspect. Preliminary studies showed the ability of LC2017 to control grapevine pathogens such as *Botrytis cinerea* (in vitro bioassays and *P. viticola* (in planta bioassays in semi-controlled conditions). Interestingly, this complex was also shown to be efficient against grapevine trunk diseases (GTDs) pathogens for which no efficient fungicide has been found since the banning of sodium arsenite [13]. GTDs, including especially Esca disease and Botryosphaeria dieback, are also currently among the most worrying diseases in vineyards because they lead to yield losses and reduce the sustainability of the vineyards [14]. Battiston et al. [12] indeed reported an in vitro growth inhibition of *Phaeoacremonium minimum*, a pathogen associated to Esca disease. In a nursery, they observed a decrease of *P. minimum* infection in propagated material treated during the hydration step. In addition, Mondello et al. [15] focused on the efficiency of LC2017 against Botryosphaeriaceae, another family of fungi related to Botryosphaeria dieback, considered as one of the three main GTDs with Esca disease. They highlighted also interesting effects of LC2017 treatments: (i) a fungistatic effect in vitro against *Diplodia seriata* and *Neofusicoccum parvum*, and (ii) in planta under controlled conditions, a weak decrease of the size of necrosis after artificial infection by pathogens. Finally, both studies reported an induction of some plant defense responses when treated by LC2017 [12,15]. Finally, the effect of LC2017 combined with the biocontrol agent, *Trichoderma atroviride* strain I-237 has been evaluated to fight *Lasiodiplodia* spp., pathogens belonging to the Botryosphaeriaceae family [16]. They preliminary concluded a promising long-term approach to mitigate the impact of Botryosphaeriaceae dieback by validating the fungistatic effect of LC2017 and the decreasing of the necrosis size after artificial infection.

Although promising for the possible use of LC2017 as a PPP to control GTDs, the results achieved so far do not give any indication on its effectiveness in controlling the expression of GTDs’ foliar symptoms in vineyards with naturally infected vines. This lack of information is essentially due to the difficulties in rapidly reproducing GTD foliar symptoms under controlled conditions [17]. The aim of the present study was thus to evaluate the ability of this copper product LC2017 on the incidence of Esca complex (including the syndromes of grapevine leaf stripe disease (GLSD) and apoplexy form) in established vineyards. To decipher possible activity mechanisms, the impact of this formulation was also investigated on the vine microbiome, vine physiology and on the enological parameters of juice. 

## 2. Materials and Methods

### 2.1. Experimental Conditions

#### 2.1.1. Plot

**Figure 1:**
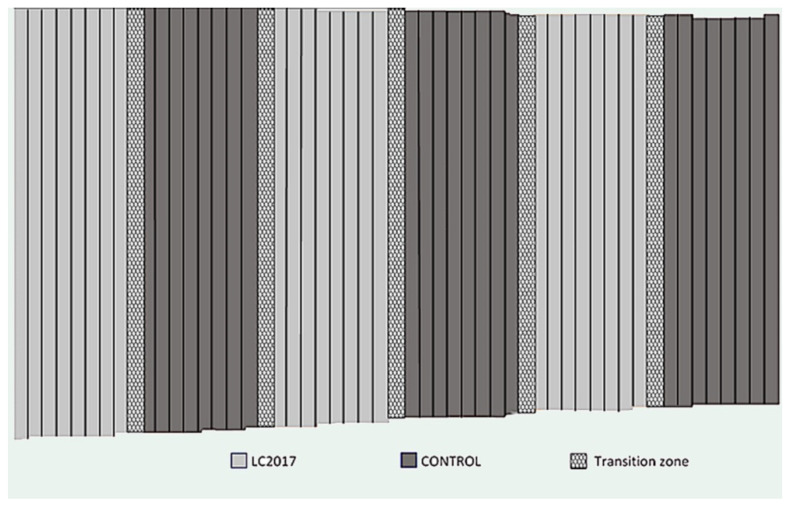
Scheme of the vineyard cv Chardonnay used from 2015 to 2019 to test the effect of LC2017 product on Esca disease control. Vines in transition zone were not considered for analysis (approximately 121 to 135 vines/row).

The experiment was carried out from 2015 to 2019 in a vineyard of the Champagne region (Avize, Marne, 48°58′48.4″ N 4°00′27.2″ E). Vines were planted in 1997 with cv Chardonnay grafted on 41B rootstock. They are pruned using the Chablis system. The soil is clay and sandy loam. A vineyard with 0.75 ha surface of the vineyard, corresponding to approximately 5200 plants, was divided in six plots, which were alternately treated with formulated HA+Cu(II), namely LC2017, and untreated, namely Control (Figure 1). Control and LC2017 treated plots are each composed of 2500 vines.

#### 2.1.2. LC2017 Product Formulation and Field Treatments Schedule

LC2017, developed and formulated by the Natural Development Group company (NDG, Castelmaggiore, Italy), contains a low amount of copper (35 g Cu^++^·L^−1^) linked to a carrier molecule (10% *v*/*v*), a biomimetic nanostructured form of hydroxyapatite (HA) (Microsap^®^—patented by NDG; [18]. HA is able to control not only the copper release but also its persistence on treated plants [12,13,14,15]. 

From 2015 to 2017, a set of preliminary assays (four treatments in 2015 and 2016 with a maximum 250 L∙ha^−1^ of water per treatment, five treatments in 2017 with a maximum 250 L∙ha^−1^ of water per treatment) focused on defining the frequency of treatments, the timing of sprays and the product/water ratios to determine a final protocol in 2017 (Table 1). The LC2017 treatments protocol consisted in five sprays per year according to vine’s phenological stages, namely immediately after winter pruning, at winter bud stage (BBCH 00, BBCH i.e., Biologische Bundesantalt Bundessortenamt und CHemische industrie [19]), at four leaves separated (BBCH 14), buckshot berries (BBCH 73), at pre-veraison (BBCH 81-83) and at maturity (BBCH 97-99) after grape harvest. Two LC2017 concentrations were used: 0.6% in the first two treatments (BBCH00 and BBCH14) and 0.5% for the last three (BBCH73, BBCH 81-83, BBCH97-99). To ensure the optimal crop coverage and to avoid product wastefulness, different volumes from 250 to 400 L·ha^−1^ were sprayed for the five treatments, in agreement with the vines canopy development (Table 1).

#### 2.1.3. Field Surveys and Esca Incidence Analysis

**Figure 2:**
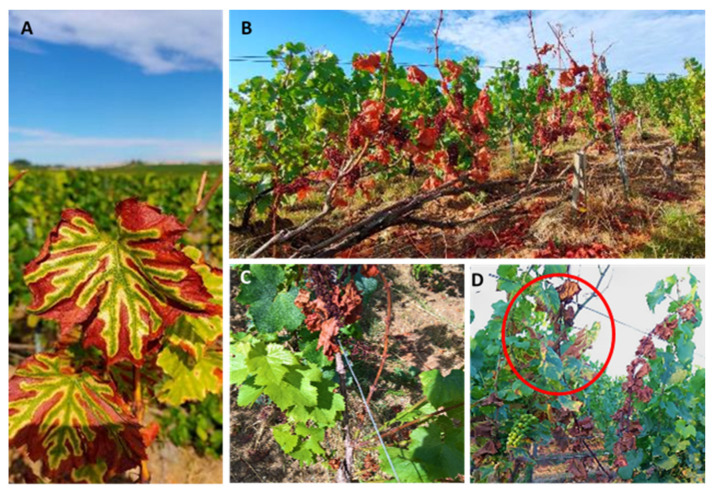
Esca symptoms observed in cv Chardonnay during vineyard surveys: (**A**) the typical tiger-striped leaves (GLSD) of Esca-affected vine; (**B**) a complete apoplectic vine with mummified grapes; (**C**) apoplectic cane beside other healthy (partial apoplexy) within the same vine; (**D**) a vine showing contemporary GLSD (red circle) and apoplexy symptoms.

From 2015 to 2017, annual surveys were carried out at harvest (end of August–September) for Esca foliar symptoms, both chronic (GLSD, Figure 2A,D) and severe (apoplexy—Figure 2B,C) [13]. From 2018 onwards, vines were analyzed three times per year for Esca symptoms, from pea-sized berries to harvest. Data related to the Esca presence and symptom severity were reported on a map of the vineyard, for each individual vine. These data were useful to determine which vines could be considered not affected by Esca disease and its spread in the studied vineyard over the years. Thus, both annual (ratio between symptomatic plants of the year and the total observed) and cumulative (ratio between symptomatic plants that showed symptoms the previous year added to those showing symptoms in the year of survey and the total observed) Esca incidence were calculated. Vines from the transition rows between Control and LC2017 plots (i.e., buffer zones) were not considered for the calculation. Esca cumulative incidence data were subject to statistical analysis by using a Mann–Whitney non-parametric test. For annual incidence, we decided to only focus on the trend and did not perform statistical analysis related to the erratic expression of foliar symptoms.

#### 2.1.4. Whole Vines Sampling

**Figure 3:**
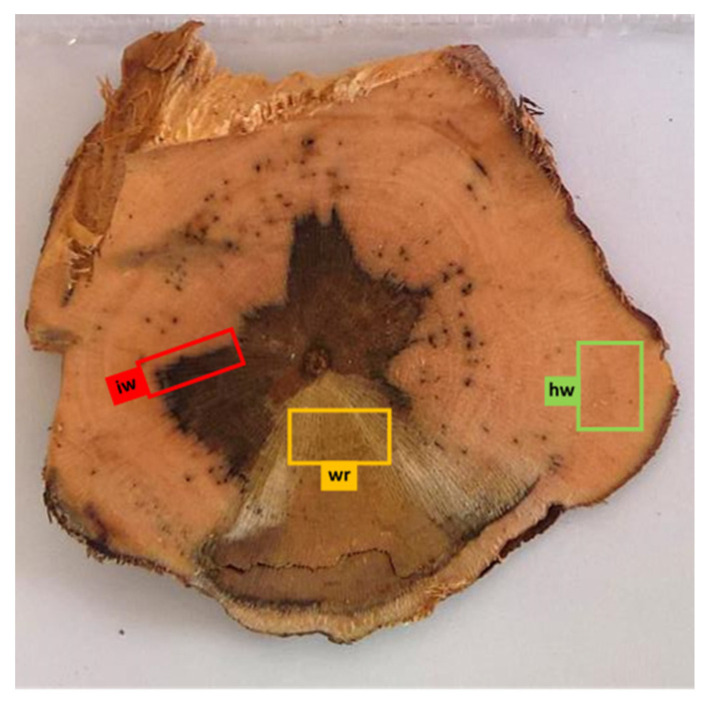
Internal symptoms in trunk section of vine cv Chardonnay affected by Esca disease and treated with LC2017. From each woody sample collected in 2019, the subsamples’ interaction zone (iw, in red), the healthy wood (hw, in green) and the white rot (in yellow, if present) were used for metabolomic (iw, hw) and microbiome (iw, hw and wr) analyses.

At 2019 harvest, 15 vines belonging to three different conditions were identified and then uprooted for further analysis (Table 2). A total of five control plants were chosen among the non-treated vines that never expressed Esca symptoms since 2014 (H), five among the non-treated and symptomatic plants in 2019 for Esca chronic form (DS) and five among LC 2017-treated, asymptomatic in 2019 but symptomatic in the two previous years (DAT) plants. Asymptomatic (la), and GLSD leaves (ls) and canes (c) were collected before uprooting. They were frozen in liquid nitrogen and stored at −80 °C until transcriptomic (Tr) and metabolomic (Me) analyses. Bunches were also collected before uprooting and were gently put in plastic bags and stored in a thermic box at 4 °C. In the lab, vines were photographed and sectioned to collect the woody subsamples (Figure 3): healthy wood (no GTD symptoms—hw), interaction zone wood (area between healthy and GTD symptomatic wood—iw) and white rot (wr), if present. For each sample at least 2 g of wood was collected and stored at −80 °C. Contemporary, berry samples were manually pressed, and the juice (j) stored in 15 mL tubes at −20 °C. 

### 2.2. Analysis of the Vine Microbiome

#### Subsamples’ Preparation

To evaluate the effect of LC2017 on the vine’s woody tissues’ associated microbiome in 2019, four subsamples from each of the three conditions H, DS and DAT were considered for analysis, namely canes (c), healthy wood (hw), interaction zone wood (iw) and white root (wr). A total of five biological replicates were considered for each condition and subsample (Table 2). A total of 55 samples were then ground to obtain a woody powder and 25 mg of each sample was then used for DNA extraction by using the Danagene Microbiome Soil DNA Kit from Danagene. 

Both bacterial and fungal microbial communities associated with vines’ woody tissues were analyzed and the 16S rRNA and ITS marker regions were selected, respectively. Samples were analyzed for the 16s rRNA V4 region and for the ITS region, using WineSeq^®^ custom primers accordingly to the Patent WO2017096385 [20]; then each library was pooled in equimolar amount and sequenced on an Illumina MiSeq instrument (Illumina, San Diego, CA, USA) using paired-end 2 × 301 paired-end reads, according to the Biome Makers protocol. 

The bioinformatic processing of reads was performed through a QIIME-based custom bioinformatics pipeline (Patent WO2017096385). A first quality control was used to remove adapters and chimeras [21]. After that, reads were trimmed out from when they did not reach the appropriate quality parameters and then the operational taxonomic units (OTU) clusters were performed using 97% identity. Taxonomy assignation and abundance estimation were obtained comparing OTU clusters by using the SILVA database, version 132 [22] and UNITE database version 7.2 [23] as taxonomic references and for bacterial and fungal microorganisms, respectively. 

Samples were analyzed for the Alpha- and Beta-diversity separately and by using the R package vegan [24] and using the OTUs counts. Regarding the Alpha-diversity, Shannon’s index and observed richness were calculated and plotted against the conditions in analysis (treatment and wood type) and by using a two-way ANOVA test (*p* < 0.05) and Duncan post-hoc analysis. The beta-diversity was analyzed through a Kruskal–Wallis non-metric multidimensional scaling in conjunction with Aitchison distances.

### 2.3. Metabolomic Analysis

Samples were firstly ground using a mechanical grinder (woody tissues) or mortar (leaves) with liquid nitrogen, and the respective powder was stored at −80 °C. For each sample, 15 mg of powder was suspended in 1 mL methanol HPLC-grade (Fluka Analytical), placed in a refrigerated ultrasonic bath for 30 min and centrifuged at 25,000× *g* for 10 min. The supernatant (1 mL) was put in 1.5 mL vials and sent to the lab for FTICR-MS analysis where it was re-diluted in methanol (1/50 [*v*/*v*]). Ultra-high resolution mass spectra were acquired using an Ion Cyclotron Resonance Fourier Transform Mass Spectrometer (FT-ICR-MS) (solariX, Bruker Daltonics GmbH, Bremen, Germany) equipped with a 12 Tesla superconducting magnet (Magnex Scientific Inc., Yarnton, UK) and an APOLO II ESI source (Bruker Daltonics GmbH, Bremen, Germany) operated in the negative ionization mode. Samples were introduced into the microelectrospray source at a flow rate of 120 µL·h^−1^. Spectra were acquired with a time domain of 4 mega words over a *m*/*z* mass range of 100 to 1000, and 300 scans were accumulated per sample. 

MS data (*m*/*z*) were submitted to NetCalc algorithm to get compound elemental chemical formula and to Masstrix (http://masstrix3.helmholtz-muenchen.de/masstrix3/, accessed on 8 October 2020) to obtain identification against *Vitis vinifera* databank (negative mode, 1 ppm error). The list was, thereafter, manually curated to remove isotopic forms (^15^N, ^18^O) and to revised wrong formula assignations. Statistical analysis was performed with the Perseus software (http://www.perseus-framework.org, accessed on 2 October 2019) that allowed us to identify significant m/z per conditions with multivariate ANOVA analysis (*p* < 0.05). In a global approach encompassing all organs (leaves, canes, woods (two zones) and grapes (juice), the list of *m*/*z* was reduced to organ-significant ones and used to generate hierarchical clustering (HCA). In a second approach, the dataset list was split per organ in order to determine symptoms-*m*/*z* discriminant (*p* < 0.05). Those organ subsets of *m*/*z* were then used to build principal component analysis (PCA) (RStudio (http://www.rstudio.com/, accessed on 28 August 2019) and Venn diagram. For specific sample comparison, elemental formula were classified into putative chemical functional categories (lipids, peptides, amino sugars, carbohydrates, nucleotides and phytochemicals/aromatic compounds) using the multidimensional stoichiometric constraints classification (MSCC) described by Rivas-Ubach et al. [25]. “Phytochemicals” was used for “Phytochemicals/Aromatic compounds” which corresponds to secondary metabolites.

### 2.4. Transcriptomic Analysis

Asymptomatic (H-la, DS-la, DAT-la) and GLSD (DS-ls) leaves (three replicates for each subsample), previously grinded in liquid nitrogen, were used to evaluate the possible elicitor effect of LC2017 on genes related to the vine defense system. Following RNA extractions, quantitative Real-Time RT-PCR analysis were performed according to Magnin-Robert et al. [26] and Spagnolo et al. [27], respectively. Results were expressed as the values of relative expression (ΔΔCt), corresponding to the mean of the three independent biological replicates. The targeted genes were selected based on similar previous studies [26,27,28,29,30,31]. Twelve genes were chosen to evaluate the grapevine response to LC2017 treatments. Among these, some genes were linked to the phenylpropanoid pathway (*PAL* and *STS*) and other defense protein markers (*CHIT4C*, *GLUC*, *PR1* and *PR10*), to detoxification processes (*GTS1*), to photosynthetic activity (*PsbP1* and *RbcL*) and to the “recovered” health-status markers highlighted in leaves of GTD-infected vines treated with sodium arsenite (*MSR*, *WRKY* and *HYD2*; Fontaine F. unpublished data) (Supplementary Table S1). Genes studied were considered up- or down-regulated when changes in their expression were either >2-fold or <0.5-fold, respectively.

### 2.5. Juice Analyses

Juices stored at −20 °C were analyzed (T = 20 °C) for classical enology parameters such as the pH, sugar concentrations, total acidity (TA) and ammoniacal nitrogen, and with an enzymatic kit (Biosentec) for α-amino nitrogen by using FTIR (OenoFossTM). For each of the three vine conditions (H, DS, DAT), two grape juices per plant (five plants per condition, except for the H condition, where one vine did not have any grape berries) were analyzed and results were expressed as averages over the two biological replicates with standard deviation.

## 3. Results

### 3.1. Effect of LC2017 Treatments on Esca Disease Symptoms Expression

**Figure 4:**
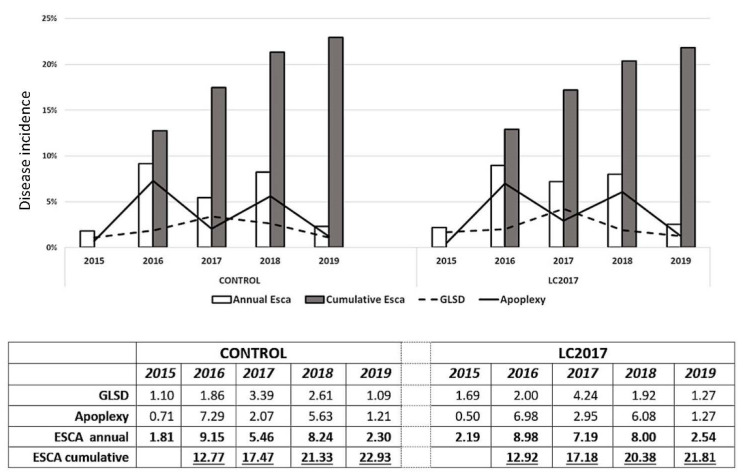
Percentages of Esca incidences (annual, cumulative, grapevine leaf stripe disease (GLSD) and apoplexy) recorded in the vineyard cv Chardonnay from 2015 to 2019 2019 in both LC2017-treated and control plots.

Results of the Esca surveys carried out from 2015 to 2019 are reported in Figure 4. Even if LC2017 treatments of the first three years differed slightly from the protocol applied in 2018 and 2019, their potential effect on Esca disease was also considered in this study. 

#### 3.1.1. Annual Incidence

**Figure 5:**
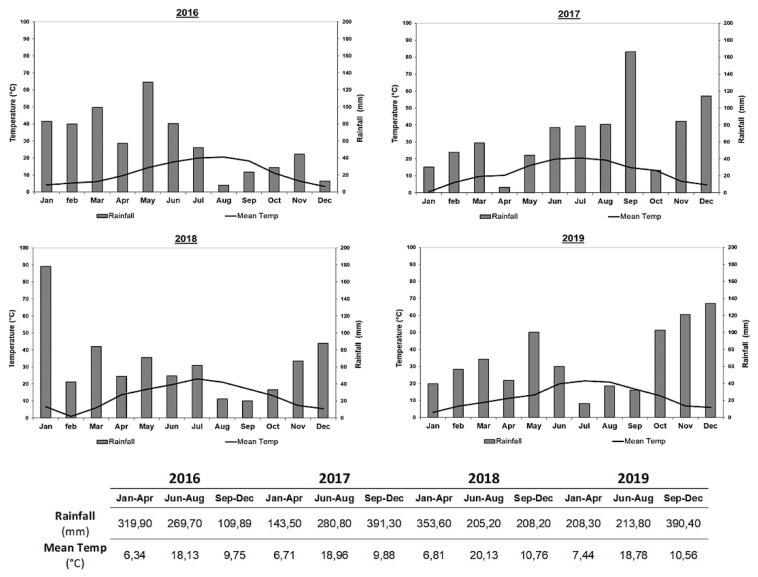
Mean temperature (left Y axes, in °C) and rainfall (right *Y* axes, in mm) recorded in the study site each year of study (*X* axes) from 2016 to 2019. The ombrothermic diagrams show the monthly rainfall (bars) and the mean temperature (lines). In the table below, values are grouped in periods of 4 months.

At the first annual survey (2015), the typical Esca symptoms expression, GLSD (Figure 2A) and apoplexy (Figure 2B,C), were observed and they were sometimes within the same plant (Figure 2D). From 2015 to 2019, Esca annual incidence values showed variations between years for control plots, ranging from 1.81% in 2015 up to a maximum in 2016 with 9.15% and then decreased slowly to reach 2.30% in 2019 (Figure 4). A similar trend with annual fluctuation for the annual incidence is recorded for the LC2017 treated plot. Regarding monthly climatic parameters in the studied vineyard, rainfall and mean temperature, there does not seem to be evidence of any particular relationship with the Esca annual incidence (Figure 5). The highest mean temperature (20.13 °C) and the lowest rainfall (205.20 mm) characterized the climate of the period Jun–Aug 2018 during the 4 years for an annual disease expression of 8.2% (Figure 4). This was opposite to the same period in 2016 with lower mean temperature (18.13 °C) and higher rainfall (269.70 °C) for a higher annual disease expression reaching 9.15% (Figure 4 and Figure 5). Conversely, rainfalls were higher in both winter and spring periods in 2016 than in 2018. In our study, the recorded Esca annual incidence values were not affected by the LC2017 treatments. The well-known erratic behavior of foliar symptom expression in Esca-diseased vines might have masked the effects of LC2017 in the treated plot. 

#### 3.1.2. Cumulative Incidence from 2015 to 2019

The count of the new symptomatic plants over the years led us to obtain a more realistic evaluation of the Esca spread in the vineyard and on the effect of LC2017 treatments on its expression. The first data on Esca cumulative incidence (2016, Figure 4) showed a uniform expression, with almost similar values between control and LC2017-treated plots (12.77% and 12.92%, respectively). This uniformity changed progressively over the 5-year survey period: in 2019, the cumulative Esca incidence reached 22.93% in the control plots while it was 21.81% in the treated plots. Contrary to annual incidences, in which the year’s higher values were recorded either in treated or control plots, the cumulative Esca incidence values since 2017 have always been higher in controls, with increasing differences from those of treated plots. In 2019, this difference reached 1.12% and was not related to less GLSD or apoplexy or both but to one or the other depending on the year (less GLSD in 2018, less apoplexy in 2017 and 2019). Despite the decreasing trend for the cumulative ’incidence in treated plot, the statistical analysis did not evidence significant differences. By contrast with the annual values, those related to the cumulative Esca somehow indicate a decrease of symptomatic vines only in the treated plot. Therefore, it is conceivable that, overall, the 5 years of LC2017 treatments had an effect in limiting the symptom expression, through its positive effects on vine physiology, in particular on photosynthetic activity and induction of plant defense genes, as observed in our previous greenhouse studies [15].

#### 3.1.3. Grapevine Leaf Stripe Disease (GLSD) vs. Apoplexy

**Figure 6:**
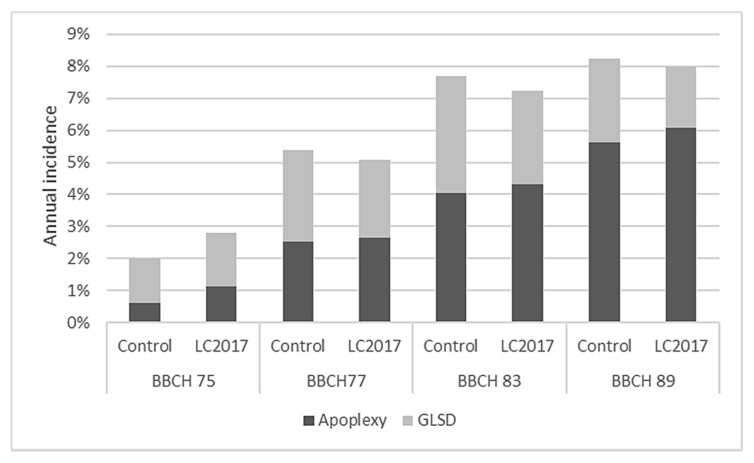
Results of the Esca symptoms (GLSD and apoplexy) surveys carried out during the whole summer in 2018 in both LC2017-treated and control plots. BBCH is phenological scale set up from the Biologische Bundesanstalt, Bundessortenamt und CHemical industry. BBCH 75 = Berries pea-sized, bunches hang, BBCH 77 = Berries beginning to touch, BBCH 83 = Berries developing colour, BBCH 89 = Berries ripe for harvest.

A different contribution of GLSD and apoplexy symptoms in the annual incidences was also observed. In 2016 and 2018, when Esca annual incidence in both control and treated plots reached or exceeded 8%, the apoplexy was the main Esca symptom expressed, contributing up to 87% in the total annual Esca incidence. For the aforementioned two years, the incidence of the apoplexy was higher in treated vines than in control (Figure 4). On the contrary, in years with lower disease expression (2015 and 2019), the main symptom was GLSD, which contributed at least up to 70% of the annual Esca incidence values. For these two years, the incidence of the apoplexy was also higher in treated plots than in control ones (Figure 4). The monthly surveys carried out in 2018 and 2019 also allowed us to assess both appearance and progression of Esca symptoms during the vegetative season. Those performed especially in 2018, with a high level of Esca expression, indicated that this expression in both treated and control plots was in early summer (June) mainly for GLSD, which reached the maximum expression in July (Figure 6). By contrast with GLSD, apoplexy incidence values increased all along the monthly survey period, probably due to the increasing need for water by plants, with higher values always recorded in LC2017 plots, as also observed in 2016 and 2017.

Globally, LC2017 treatments did not induce a significant reduction of Esca symptoms expression for affected vines, even if constant reductions of cumulative incidence values between treated and control plots were reported. This reduction was related to the lower presence of GLSD symptoms in treated vines compared to those recorded in control (Figure 4 and Figure 6). The reduction in GLSD and not in apoplexy could be due to the different origins of these symptoms. It is thus hypothesized that GLSD is mainly due to fungal phytotoxins accumulation in leaves whereas apoplexy results from dysfunction of the vine vascular system. LC2017 could thus have an effect limited to GLSD, maybe by limiting fungal toxin accumulation in leaves. This would merit being confirmed.

### 3.2. Impact of LC2017 on the Vine Microbiome 

**Figure 7:**
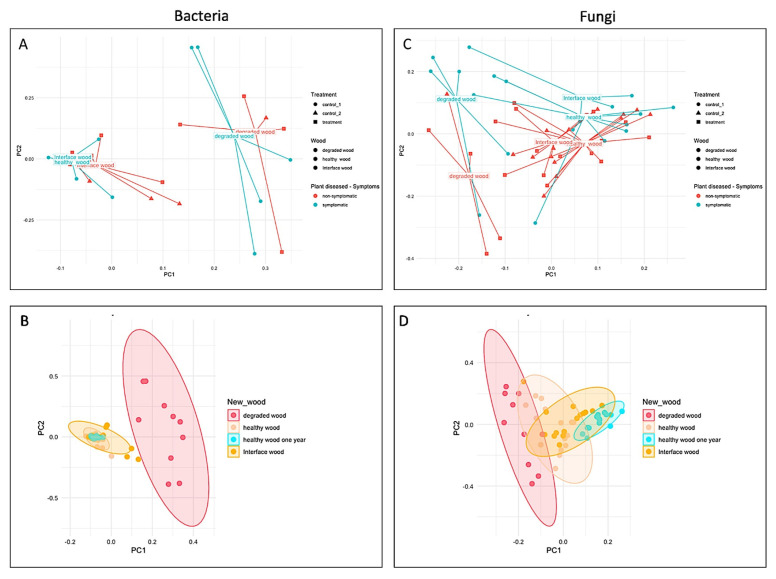
Beta-diversity of bacterial (**A**,**B**) and fungal population (**C**,**D**) according to the treatment (LC2017, corresponding to DAT; control 1 (symptomatic, corresponding to DS) and control 2 (asymptomatic, corresponding to H)), wood type zone (white rot = degraded wood; health wood; interaction zone = interface wood) and symptoms expression.

Analysis of the beta-diversity (Figure 7) shows a clear population dynamic between the degraded (white rot—WR) and with the interface (interaction) and healthy wood. In spite this being particularly notorious for the bacterial population (Figure 7A,B), a statistical difference by wood type is observed for both bacteria and fungi (*p* < 0.001) and is, thus considered as the main driver of the beta-diversity patterns. Moreover, the microbial distribution showed that LC2017 treatment had a significant effect on fungal communities (*p* < 0.026) although this was not significant across bacterial communities (*p* < 0.36). Considering the expression of symptoms, it is possible to identify clusters concerning the expression of symptoms (symptomatic vs. asymptomatic), although no significant differences across bacterial (*p* < 0.507) and fungal communities (*p* < 0.079) were observed.

**Figure 8:**
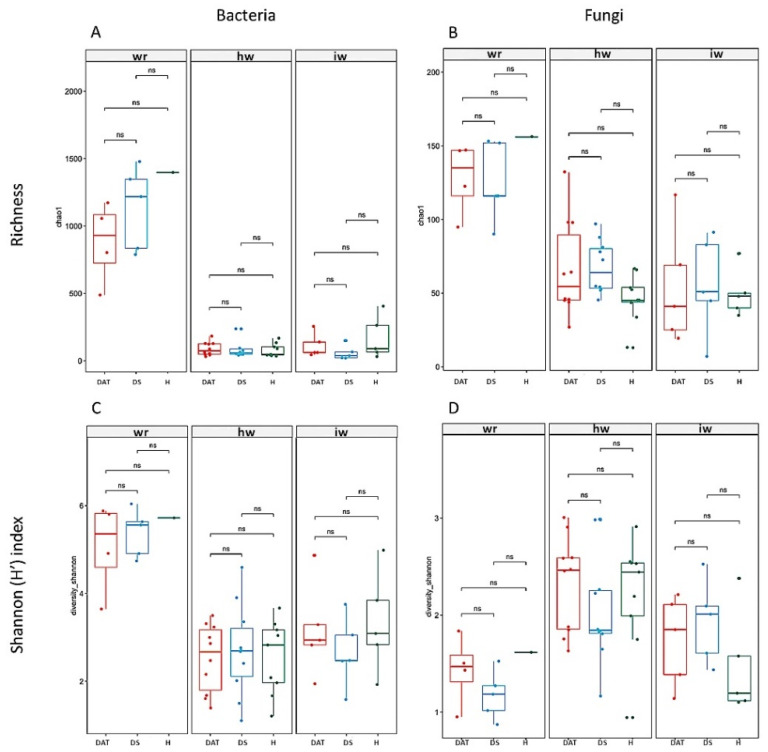
Alpha-diversity (OUT richness/Chao1 and Shannon (H’) index) of bacterial (**A**,**C**) and fungal population (**B**,**D**) according to the wood type (wr = white rot; hw = health wood; c= canes, iw = wood interaction) and condition (DAT-LC2017 treatment, DS-control 1 symptomatic and H-control 2 asymptomatic). The box limits are related to the 25th and 75th percentile and median is represented by a central line. Dots are outliers, namely points below 25th percentile—(1.5 * IQR) and above 75th percentile + (1.5 * IQR), where IQR is the interquartile range or absolute difference between 75th and 25th percentiles.

Concerning the alpha-diversity (Figure 8), the wood type (degraded, healthy and interface wood) showed a clear impact on the diversity of bacterial and fungal communities, as shown for OTUs richness and Shannon (H’) index values. Indeed, these results confirm that wood type is the main driver of changes in the alpha-diversity and that treatment did not show a significant effect on the microbial biodiversity. Regarding the bacterial communities, this population showed a higher diversity than fungal, and among it the degraded wood showed a higher diversity while healthy, and interface wood showed a similar alpha-diversity values. This trend was similar across fungal communities.

At the taxonomy level, a distinct microbial profile associated within each wood type is clearly observed for fungal population (Supplementary Figure S1) while for bacteria, a more homogenous profile is observed across wood samples (Supplementary Figure S2). Regarding the treatment effect, both control and treated samples showed a similar microbial profile for both fungal and bacterial population at the phylum and class levels, suggesting that treatments did not influence negatively the natural microbial communities. 

**Figure 9:**
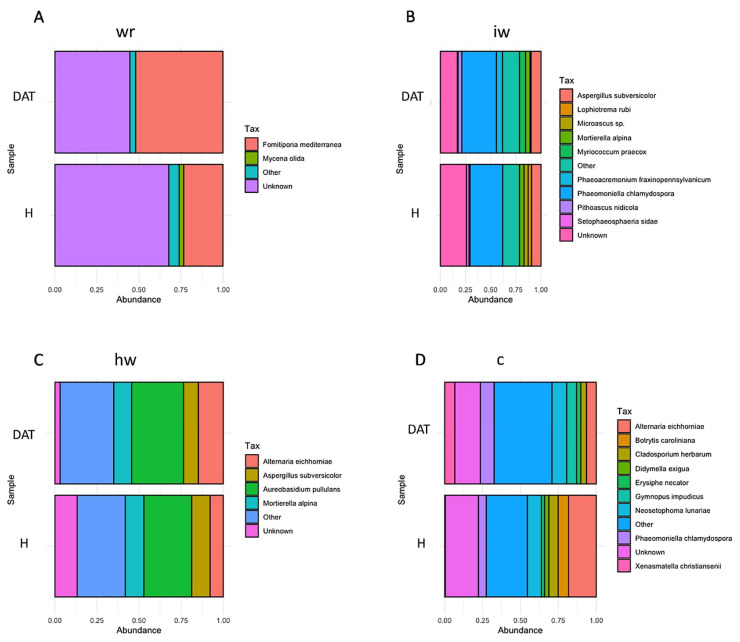
Relative abundance of the fungal species level in H (control) and DAT (LC2017-treated) wood subsamples. In (**A**), white rot; in (**B**), interaction wood; in (**C**), health wood; in (**D**), canes. wr = white rot; hw = health wood; c = canes, iw = wood interaction).

As regards the fungal communities, the degraded wood showed a very distinct profile in which the Basidiomycota was the higher phylum (Agaricomycetes class); interface wood was dominated by the Ascomycota phylum and the Eurotiomycetes class; while both heathy and healthy wood after 1 year showed a very similar profile dominated by the Ascomycota phylum and the Dothideomycetes class (Figure 9). At the species level, the degraded wood was dominated by *Fomitiporia mediterranea*, an Esca-associated pathogen [32]. The interface wood was characterized by *Phaeomoniella chlamydospora* with a similar incidence for both control and treated samples. The healthy wood was characterized by the presence of *Aureobasidium pullulans*, while the heathy wood after 1 year started to show the incidence of the pathogen *Phaeomoniella chlamydospora* with a slightly lower incidence at treated samples than at control. 

**Figure 10:**
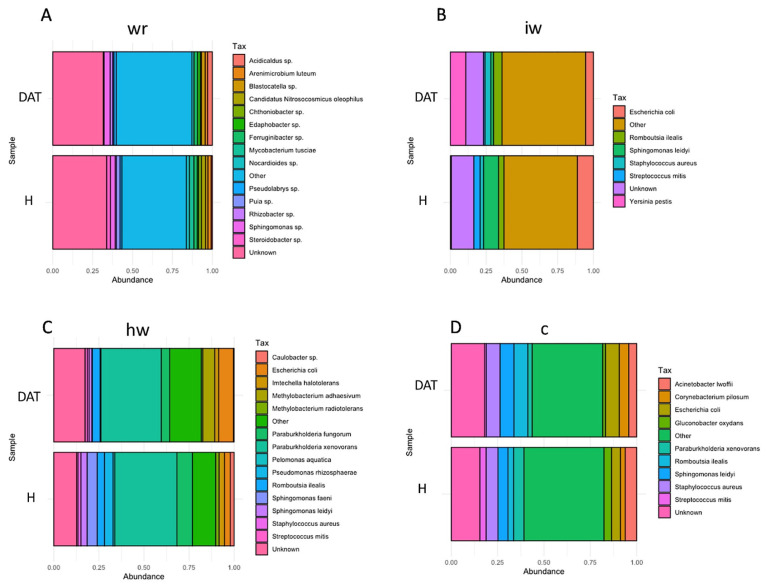
Relative abundance of the bacterial species level in H (control) and DAT (LC2017-treated) wood subsamples. In (**A**), white rot; in (**B**), interaction wood; in (**C**), health wood; in (**D**), canes. wr = white rot; hw = health wood; c = canes, iw = wood interaction).

The bacterial communities showed similar microbial patterns across the different wood types and the *Proteobacteria* phylum and the *Gammaproteobacteria* and *Alphaproteobacteria* classes were the most abundant across all samples. A similar behavior was observed at species level for treated and control samples from each wood type. Overall, the healthy wood showed a more stratified microbial profile (Figure 10). Globally, the wood from vines treated with LC2017 did not show a different microbiome profile, suggesting that the treatment may not have a negative effect on this plant structure. However, more microbiome studies are needed to infer about this harmlessness of the LC2017, in particular at foliar level, in which the product is applied.

### 3.3. Impact of LC2017 Treatment on Plant Physiology

#### 3.3.1. Metabolomic Analysis

**Figure 11:**
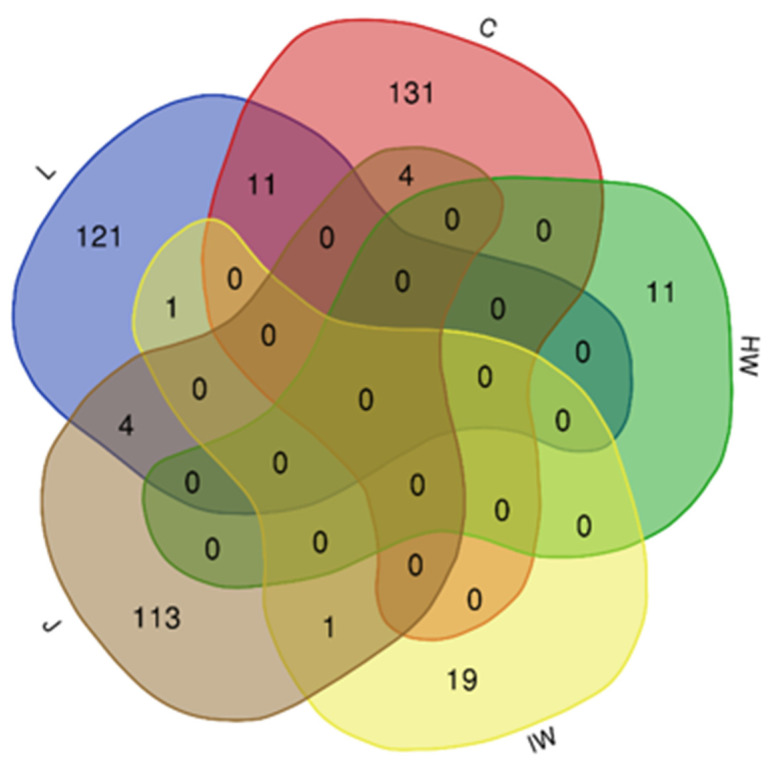
Venn diagram of symptoms discriminating significant *m*/*z* in organs/tissues. Diagrams were built from matrix reduced to significant *m*/*z* (*p* < 0.05) in canes (C, 146 *m*/*z*), in leaves (L, 139 *m*/*z*), in healthy (HW, 11 *m*/*z*) and interaction (IW, 21 *m*/*z*) woods, and juice (J, 122 *m*/*z*), that discriminate three symptom groups of vines i.e., healthy, diseased asymptomatic and diseased symptomatic.

FT-ICR-MS analysis of all samples (except woody white rot ones) provided a total of 3039 *m*/*z*. Hierarchical clustering analysis (HCA) of the data obtained highlighted 2579 significant *m*/*z*, and showed that samples are primarily grouped according to their origin (organ/tissue, juice) (Supplementary Figure S3). There was a higher number of specific *m*/*z* for canes, leaves and juice samples (131, 121 and 113, respectively), compared to wood samples (19 and 11 for iw and hw, respectively). Surprisingly, hw samples do not share any *m*/*z* with the other sample types (Figure 11). 

Data were therefore split into five subsets based on the sample origin, (corresponding to 1 990 *m*/*z* for leaves, 1 551 *m*/*z* for canes, 754 *m*/*z* for healthy wood, 751 *m*/*z* for interaction area in wood and 824 *m*/*z* for juice) in order to check if experimental modalities (H, DS, DAT) could be distinguished by metabolite profiles.

**Figure 12:**
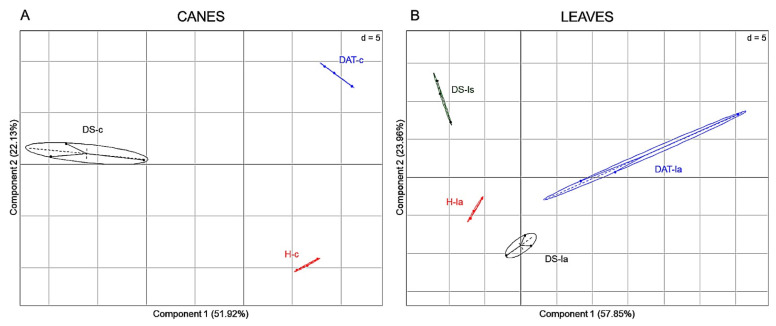
Principal component analysis (PCA) illustrations of experimental modalities in aerial parts canes (**A**) and leaves (**B**). PCA were built from matrix datasets reduced to significant *m*/*z* (*p* < 0.05), in canes (146 *m*/*z*) and leaves (137 *m*/*z*), that discriminate groups of vines (H: not-treated, healthy; DAT: treated, diseased asymptomatic and DS: untreated, diseased symptomatic) from which samples were collected. From diseased symptomatic vines, two types of leaves, i.e., asymptomatic (la) and symptomatic (ls), were collected (three replicates per group).

Canes extracts from the three modalities H, DS and DAT could be well discriminated (Figure 12A), with 146 significant *m*/*z*. In the same manner, these modalities could be distinguished in leaves (137 significant *m*/*z*), and in symptomatic and asymptomatic leaves of untreated vines expressing the disease (DS-ls and DS-la, respectively) (Figure 12B). These results thus show an impact of LC2017 treatments, and also of the fungal infection, on both cane and leaf metabolomes. Such a discrimination was also observed for wood samples, although with a lower number of discriminant *m*/*z* (11 and 21 m/z for hw and iw respectively) (Supplementary Figure S4). Although groups partially overlap for juice samples, DS and H samples can be well-distinguished. These results thus indicate that the metabolome of the organs or tissues analyzed is impacted to varying degrees by infection and/or by LC2017 treatment.

**Figure 13:**
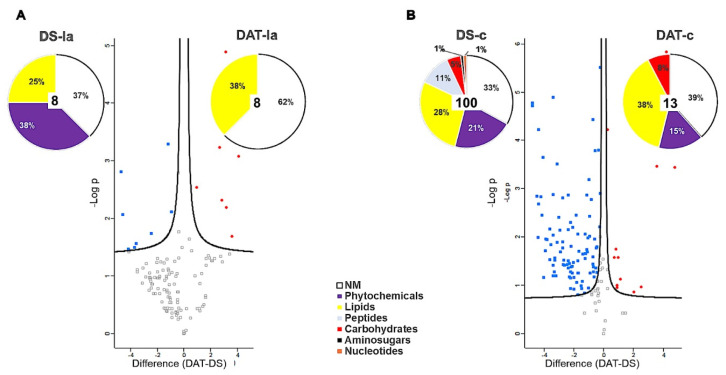
Treated (DAT) versus non-treated (DS) comparisons in asymptomatic (la) (**A**) leaves and (**B**) canes. Volcano plot representations of significant of *m*/*z* accumulated in either DS or DAT samples. Predicted structural families determined by raw formulas are illustrated on pies, indicating numbers and proportions of DS or DAT-accumulated *m*/*z* concerned.

Leaf and cane samples of LC2017-treated (DAT) and untreated diseased vines (DS) were then compared (pairwise comparison, *t*-test) to highlight differences between untreated diseased vines expressing the disease and treated ones that became asymptomatic (Figure 13). In asymptomatic leaves, 16 *m*/*z* were found discriminant to separate the two groups, of which eight were more accumulated in DAT-la samples (three lipids and five non-categorized) and eight were more accumulated in DS-la ones (two lipids, three phytochemicals, three non-categorized) (Figure 13A). Only five of them could be annotated (Supplementary Table S2) In canes, 113 *m*/*z* were differently accumulated with a higher accumulation in DS-c (100 *m*/*z*) rather than in DAT-c (Figure 13B). Only three could be annotated (Supplementary Table S3) In both DS-c and DAT-c samples, besides non-categorized metabolites (33–39% respectively), accumulated ones were lipids (28–38%, respectively), phytochemicals (21–15%, respectively), and carbohydrates to a lesser extent (5–8%, respectively). The main difference between the two groups corresponds to the accumulation of peptides in diseased DS-c samples (11%) (Figure 13B). These results thus show that LC2017 treatment of infected vines impacts the metabolic profiles of leaves and canes, according to the observation that they no longer express the disease.

Comparison was also made between leaves from apparently healthy untreated vines (H-la), and asymptomatic leaves of treated vines (DAT-la). We found 94 *m*/*z* differently accumulated: 72 in DAT-la samples and 22 in H-la samples (Supplementary Figure S5). In both sample types, most of the significant *m*/*z* were non-categorized (51% and 55% for DAT-la and H-la, respectively), and the most accumulated categorized ones were lipids (38% and 27%, respectively), then phytochemicals. The main difference was the specific accumulation of carbohydrates in H-la samples. As they could not be annotated, they are not “common” sugars known for their role in cell functioning and/or signalling in plants. We thus cannot conclude about this observation. However such a comparison shows that even if leaves of LC2017 treated vines no longer express the symptoms of the disease, their metabolome remains different from that of apparently healthy vines.

#### 3.3.2. The LC2017 Effects on the Vine Defense Responses 

**Figure 14:**
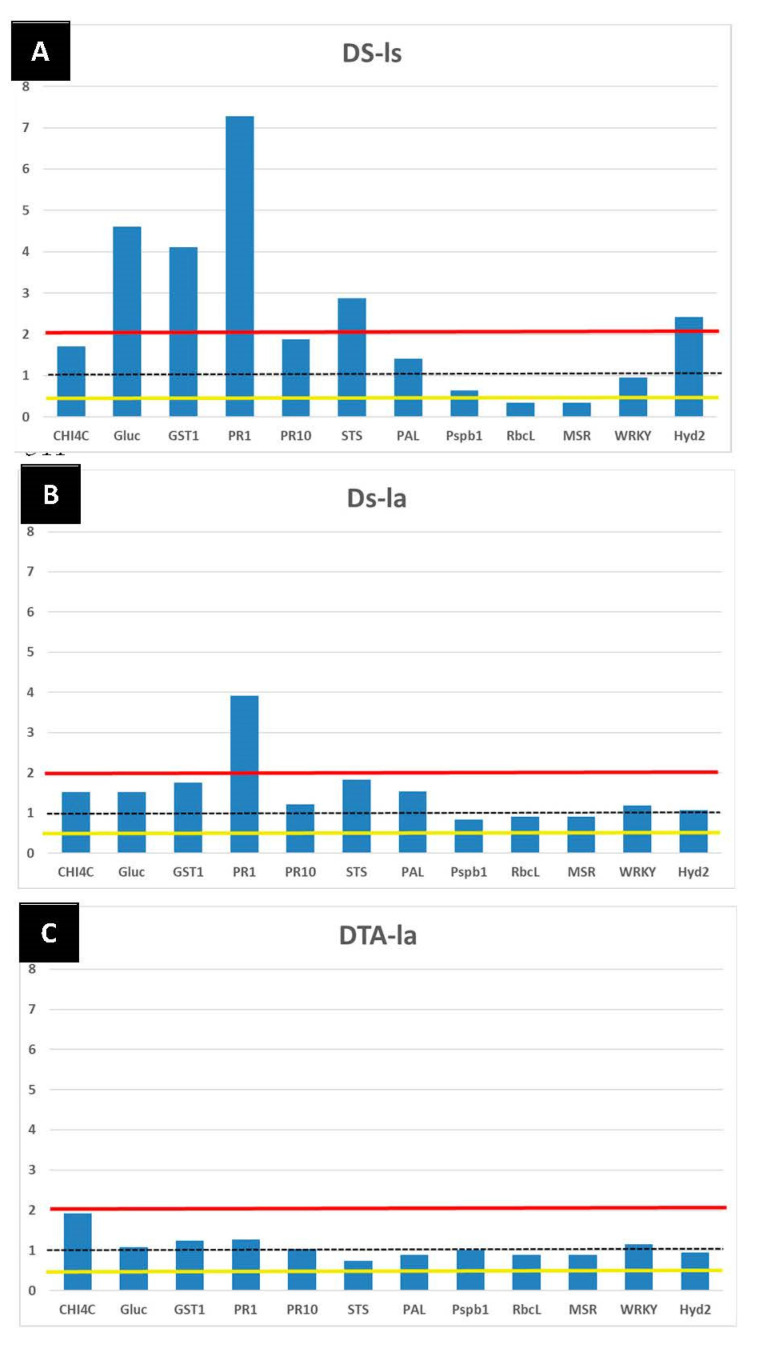
Transcriptomic profiles of the 12 targeted genes in: (**A**) GLSD (ls) and (**B**) asymptomatic (la) leaves cv Chardonnay sampled on vines belonging to symptomatic control (DS) and (**C**) LC2017 treated (DAT) vines conditions compared to the control H. Values (mean of three technical replicates) represent the expression levels (ΔΔCt) of reported conditions relative to the control (black dotted line, value = 1). Expression of a given gene was considered up- or down-regulated when value of relative expression was >2-fold (red line) or <0.5-fold (yellow line) compared to the control (H), respectively.

The study of targeted gene expression on leaves cv Chardonnay belonging to different conditions (H, DS, and DAT, see Table 2) are reported in Figure 14. Histograms show the level of expression (as folds increase compared to control H) of the 12 targeted genes in GLSD symptomatic leaves sampled from DS vines (Figure 14A) and in asymptomatic leaves sampled from DS (Figure 14B) and DAT (Figure 14C) vines, when compared to the H condition (control). The transcriptomic pattern of symptomatic leaves (DS-ls) showed several genes related to the plant disease status (Figure 14A): in particular, those related to *PR* (pathogenesis-related) proteins such as *GLUC* and *PR1*, to the phenylpropanoid pathway (*PAL* and *STS*) and to detoxification processes (*GST1*). Meanwhile, some genes were repressed, such as *RbcL* linked to the host plant photosynthetic activity. In this study, genes related to the recovered health-status observed in arsenite-treated vines were analyzed since (i) in the past this product was the sole to limit Esca foliar symptoms expression in vineyard [15] and (ii) Fontaine and collaborators (unpublished data) observed that these genes were overexpressed under arsenite treatment on diseased vines not re-expressing foliar symptoms. For the three genes considered, the expression of *HYD2* was induced while *MSR* expression was repressed in DS-ls. In asymptomatic leaves collected in GLSD vines (Figure 14B, DS-la), only the expression of *PR1* expression was induced, even if others related genes to detoxification processes (*GST1*), and phenylpropanoid pathways (*STS* and *PAL*) showed an increase in their expression. When the Esca-affected vines were treated by LC2017 (Figure 14C, DAT-la), the transcriptomic profile in asymptomatic leaves was similar to that of the control vines (DS-la), without any induced or repressed gene expression except for *PR1*. 

These results, extending the effects of LC2017 also to the host-plant defense system, strengthen the hypothesis of the “normalizing” role of LC2017 in several physiological processes in Esca-diseased plants. 

### 3.4. Impact of LC2017 on the Berry Juice 

**Figure 15:**
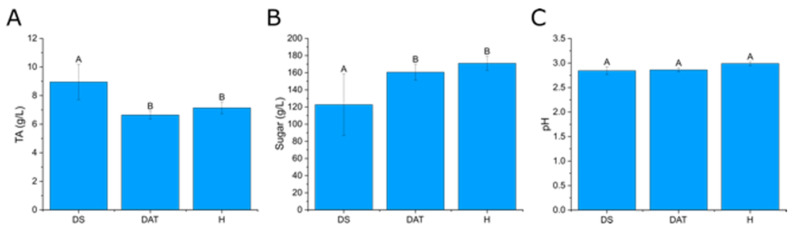
Grape juices enological parameters for the three vine conditions: Titrable acidity as g/L H_2_SO_4_ (**A**), Sugar concentration (**B**), pH (**C**); Mean values from the analysis of 2 distinct grape juices from 5 distinct vine trunks for each of the three vine conditions (only 4 vine trunks for the H condition. Different letters indicate significant differences (analysis of variance (ANOVA), Kruskall–Wallis, *p* > 0.05).

Statistical analyses (Kruskall–Wallis, *p* < 0.05) of results from the analysis of classical enological parameters (Figure 15, Table S5) revealed that significant differences could be observed between the three different vine conditions, with a lower sugar concentration and higher TA values for DS juices compared to the other juices (DAT and H). The relatively high variability between the different samples of the same vine condition can basically be explained by the natural variability which is frequently encountered among grape bunches from even the same vine trunk where, for instance, inner berries which have not experienced the same sun exposure as outer berries may exhibit significantly lower ripening levels. On average, and if considering the very low concentrations for DS4 replicates, average sugar concentrations were of the order of 120 g·L^−1^ for DS juices, whereas they were above 160 g·L^−1^ for the two other vine conditions. pH values were all between 2.8 and 3, and evidence of acidic juices. TA values appeared consistent with this observation, with averages just below 7 g·L^−1^ (H_2_SO_4_) for DAT and H conditions, and nearly 9 g·L^−1^ for DS trunks. It must be noted, however, that TA values for DS4 appeared unexpectedly high. The sum of ammoniacal and α-amino nitrogen values shows that yeast assimilable nitrogen concentrations were in general low (below 200 mg·L^−1^) and very low for DAT4 and H5 vine trunks (below 100 mg·L^−1^).

## 4. Discussion

LC2017 was assessed as a possible PPP for GTD control in the vineyard, complementing previous promising studies carried out in controlled conditions [12,15]. To confirm the interesting potential of LC2017, we analyzed its effect on the incidence of Esca, annually and cumulatively, and in parallel on the microbiome and defense responses of the vine and on the enological characteristics of the corresponding grape juices. 

The experiment was carried out in a plot in which GTD expression has been recorded since 2015, making it possible to select vines for healthy (H) and diseased (D) modalities. To date, LC2017 treatment did not show any significant impact on Esca cumulated incidence values, even if a constant low decrease was observed along the trial. This decrease in Esca cumulative incidence might suggest the activity of LC2017 on plant resilience by keeping vines as having Esca-infected status but asymptomatic. This supports the need to proceed with complementary vineyard studies in both established vineyards with similar or higher Esca disease incidences and also in young ones. Indeed, a higher disease pressure may allow better observation of the positive effects of LC2017 on limiting the disease development and on the expression of symptoms, by emphasizing the differences between treated and non-treated vines, as reported in a similar study carried out by Di Marco et al. [33]. The authors tested a copper-based product in a vineyard with higher Esca cumulative incidences (35–45%), demonstrating its efficiency in limiting GLSD symptoms after only 3 years of treatment. In parallel, treatments in young vineyards could be useful to assess the capacity of LC2017 in preserving a longer asymptomatic phase of Esca-affected vines. From 2017 to 2019, the weak increase of the apoplectic form in the treated vines might be related to the climate (rainfall, temperature) in our experimental plot since it was relatively different from January to August in 2016 and 2018, the 2 years with higher expression of the apoplexy (Figure 4 and Figure 5). The colonization history of the vines, in which several pathogens and/or more GTDs could occur simultaneously as revealed by the microbiome analysis, might have more incidence than the climate. 

The microbiome study confirms the etiology of the observed decline, by revealing the presence of the causal agents of Esca disease, namely *P. chlamydospora* and *F. mediterranea*, and to a lesser extent *Cadophora luteo-olivacea* and some *Phaeoacremonium* species in the processed wood subsamples (Supplementary Table S4). Their presence also in the wood of healthy vines (H), which newer displayed Esca foliar symptoms, confirms the well-known long asymptomatic phase. Indeed, the complexity of the pathogen-host plant interaction of GTDs is that the pathogen’s presence is not a discriminating factor between visually healthy and infected plants. Furthermore, the microbiome analysis allowed the detection of other GTD pathogens: (i) *Diplodia seriata* considered a causal agent of the Botryosphaeria dieback, (ii) several *Eutypa* species and (iii) other agents of vascular diseases such as *Fusarium* spp. This confirmed the complexity of the phytosanitary status of the trunk in established vineyards, regardless of the observable symptoms. Overall, results confirm previous studies in which no link was found between the wood microbiome and GLSD expression in vineyard [34,35,36,37]. This complex status of the wood microbiome of adult vines and the aforementioned similarity between the microbiome of symptomatic and asymptomatic vines, appears to be compatible with the hypothesis that GTD fungi can switch from a saprophytic or neutral status to a pathogenic one [36]. This pathogenic behavior could result from changes on its closed environment and may induce its production of phytotoxins [38,39,40]. This phytotoxicity could also be triggered by the alteration in the plant beneficial microbiote/microbiome. Indeed, beneficial microorganisms were recently described metabolizing/co-metabolizing the phytotoxins terremutin and mellein [31]. LC2017 could indeed interfere with phytotoxins, as reported by Di Marco et al. [33]. They observed that a copper oxychloride formulation was able to block in vitro the production of scytalone in *P. chlamydospora* and *P. minimum* and thus reduce Esca foliar symptoms in the field. However, no putative *m*/*z* corresponding to scytalone or isosclerone could be found in our FT-ICR-MS data list, making this hypothesis unable to be checked. To the best of our knowledge, our study and those of Di Marco et al. [33] are the only ones to have tested a copper-based product beyond the role of pruning wound protector for GTDs control, also verifying its effect on leaf symptom expression in the field. Regarding the bacterial part of our microbiome analysis, the presence of *Aureobasidium pullulans* in the healthy wood is notorious. This strain was first reported as a potential biocontrol agent against *Eutypa lata* [41] and, more recently against *Diplodia seriata* in an in vitro study by Pinto et al. [42]. The latter also reported the ability of this yeast-like fungi to induce host–plant defense responses at a transcriptomic level. Beside obvious bacterial and fungal population diversities observed in the different subsamples (beta-diversity), no negative effects of LC2017 treatments on the vine’s wood microbiome was detected. This is particularly important when developing new sustainable PPP, as the preservation of the balance among beneficial, neutral and pathogenic microorganisms is a key role in plant health [42,43,44,45]. However, this microbial balance is often challenged by vineyard practices and PPP [46], especially copper-based ones, as they can modify leaf, berries and soil microbiome [47].

The impacts of LC2017 on both transcriptomic and metabolomic profiles in canes and leaves were also studied to evaluate the LC2017 effect on vine physiology. As expected, genes related to the plant defenses were induced in symptomatic and untreated leaves (DS-ls) (Figure 14), in agreement with previous studies [12,15,26,27,28,48]. Such responses were thus related to the disease emergence. Among these, some are linked to cells’ detoxification (*GST1*) active in the presence of toxic metabolites [31,48] and others are pathogenesis-related protein genes (*PR1, GLUC*). Interestingly, the expression of the targeted genes is similar in leaves from healthy ones (H), asymptomatic leaves from diseased vines (DS-la) and Esca-affected treated vines (DAT), characterized by no alteration (Figure 14). To recapitulate, the LC2017 treated vines were asymptomatic in 2019, the year sampled, but were symptomatic in the two previous years. The fact that the LC2017-treated vines showed no Esca symptoms in 2019 and had a similar genes expression pattern to a healthy vine suggest a recovered/resilient status of the diseased vines related to LC2017 treatment. This recovered status of the Esca-affected plants treated with LC2017 was previously observed [49] in leaves of vines treated with sodium arsenite, now forbidden but able to control the emergence of Esca foliar symptoms in GTD-infected plants. This result could be related to the lower incidence of GLSD symptoms observed in the treated plots, thus strengthening the hypothesis that LC2017 eventually enlarges the asymptomatic phase of Esca-infected vines. 

HCA analysis of the whole metabolomic dataset straightforwardly discriminated the sample types (organ/tissue/juice) rather than the three modalities studied (H, DS, DAT). Thanks to analyses performed for each sample type data set, we reported a differential accumulation of metabolites between the three modalities, especially in leaves and canes, but also in wood samples. Leaves and canes directly received the LC2017 treatment, and we can thus assume that the treatment sustainably impacted their metabolome. As canes are an area of sap fluxes, we can also assume that they reflect changes occurring both in leaves (via phloem sap) and wood (via xylem sap). LC2017 treatment is carried throughout the plant by hydroxyapatite, and it might thus directly reach woody tissue and affect its metabolome. However, it is important to keep in mind that the detected metabolites can be either vine metabolites, vine metabolites modified by microorganisms, and/or microbial metabolites. LC2017 treatment might also indirectly impact the wood metabolome through its effects on the vine microbiome (described above) and related metabolism. When comparing DAT to DS samples, only few discriminant *m*/*z* were found in leaves, making it difficult to discuss their role. More discriminant *m*/*z* were obtained in canes, among which a higher percentage of lipids than phytochemicals in diseased asymptomatic treated (DAT) samples than in diseased symptomatic (DS) ones. Interestingly, similar observations were made when comparing the metabolome of the brown stripe symptomatic area and adjacent asymptomatic one of Botryosphaeriaceae infected trunks [50].

The differences between the modalities became less evident for the metabolites of juice samples, which partially overlap, suggesting limited effects of the treatment on the grape metabolome. This might be due to the fact that the inflow of xylem sap towards berries decreases or ceases after veraison, while the inflow of phloem sap increases until near harvest [51]. Thus, berries would not be—or would be much less—impacted by metabolic changes occurring in woody tissues, contrary to what has been suggested for canes, hence a lesser effect of LC2017 treatment on juice compositions. However, classical enological analyses revealed some significant differences between conditions, with a lower sugar content and higher total acidity in DS juices, compared to DAT and H ones. This result, that agrees with other studies [52,53] which observed differences in the musts composition from Esca-affected and healthy vines, suggests a hindering of the plant metabolism for diseased trunks that would be compensated for by the LC2017 treatment. It must be noted that the corresponding low pH values could be explained by the fact that vine trunks, to avoid the overlapping of eradication and harvest, were unrooted 7 days before harvest. Finally, some values (in particular for DS4 and H2, Supplementary Table S5) appeared to be out of the normal range, which could be explained either by an analytical bias or by some matrices clearly different from reference matrices used for FTIR calibration. For the DS4 sample, this latter explanation is supported not only by the very low sugar concentrations, but also by the ammoniacal nitrogen value. Enzymatically measured α-amino nitrogen values seem to further confirm the outlier character for DS-j (sample 4) and H-j (sample 2), indicating a likely high heterogeneity among vine trunk metabolisms. Nevertheless, yeast assimilable nitrogen also suggested must deficiency in general for these vines, regardless of the vine condition. 

Globally, the study on the effect of LC2017 carried out in established vineyards to control Esca disease confirmed its promise as previously reported [12,15,17] in a context of GTD diebacks. 

## 5. Conclusions

The present study showed a trend of decreasing Esca foliar emergence over the years and restoration of a healthy status since the vine physiology (metabolomic and transcriptomic) in leaves of diseased LC2017-treated vines was similar to that in the leaves of healthy vines. In addition, no relevant impacts on the enological quality of berries and on the vine microbiome were observed. These outcomes encourage the need for further studies using LC2017 as a PPP to control GTDs, testing it on other cultivars more susceptible than Chardonnay to GTDs.

## Figures and Tables

**Table 1:** Protocol used for the LC21017 treatment applied since 2018.

	1st Treatment	2nd Treatment	3rd Treatment	4th Treatment	5th Treatment
	BBCH 00	BBCH 14	BBCH 73	BBCH 81-83	BBCH 97-99
LC2017	1.5 L·ha^−1^	1.5 L·ha^−1^	2 L·ha^−1^	2 L·ha^−1^	2 L·ha^−1^
Water	250 L·ha^−1^	250 L·ha^−1^	400 L·ha^−1^	400 L·ha^−1^	400 L·ha^−1^
LC2017 final concentration	0.6%	0.6%	0.5%	0.5%	0.5%

**Table 2:** Terminology used the vineyard sampling carried out in 2019 to evaluate the effects of LC2017 in standing vines cv Chardonnay belonging to three different conditions: control plants asymptomatic (H) and symptomatic (DS) and LC2017-treated plants asymptomatic (DAT). For each condition, the sub-sample type with the related codes (capital letter = vine condition, minus letters = subsample), the kind of analysis (Me = metabolomic, Mi = microbiome, Tr = transcriptomic) and, in brackets, the number of subsamples processed out of the five available are recorded.

Vine Condition	Vines n.	Subsample						
		Leaves		Canes c	Grape Juice j	Healthy Wood hw	Wood Interaction Iw	White Rot Wr
		*Asymptomatic* la	*Symptomatic* ls					
Healthy not treated H	5	H-la		H-c	H-j	H-hw	H-iw	H-wr
		Me(3)–Tr(3)		Me(3)–Mi(5)	Me(5)	Me(3)–Mi(5)	Me(3)–Mi(5)	Mi(1)
Diseased symptomatic not treated DS	5	DS-la	DS-ls	DS-c	DS-j	DS-hw	DS-iw	DS-wr
		Me(3)–Tr(3)	Me(3)–Tr(3)	Me(3)–Mi(5)	Me(5)	Me(3)–Mi(5)	Me(3)–Mi(5)	Mi(5)
Diseased asymptomatic treated DAT	5	DAT-la		DAT-c	DAT-j	DAT-hw	DAT-iw	DAT-wr
		Me(3)–Tr(3)		Me(3)–Mi(5)	Me(5)	Me(3)–Mi(5)	Me(3)–Mi(5)	Mi(4)

## Data Availability

Not applicable.

## Supplementary Materials

The following supporting information can be downloaded at: https://www.mdpi.com/article/10.3390/jof8020151/s1, Figure S1: Relative abundance of the fungal phylum class level across control and treated samples (LC2017) and concerning the wood types in analysis; Figure S2: Relative abundance of the bacterial phylum class level across control and treated samples (LC2017) and concerning the wood types in analysis; Figure S3: Hierarchical clustering (HCA) of significant metabolites discriminating organs/tissue; Figure S4: Principal component analysis (PCA) illustrations of experimental modalities for healthy (hw) and interaction area (iw) of would samples, and juice (j); Figure S5: Comparison of asymptomatic leaves sampled from LC2017 treated (DAT) and healthy (H) vines. Table S1: List of the genes targeted in the transcriptomic study; Table S2: Identified asymptomatic Leaves metabolites from treated and non-treated vines (DAT-la) versus (DS-la). From 16 significant *m*/*z* (*P*: ANOVA *p* value) discriminating the two conditions DAT and DS asymptomatic leaves (*see* Volcano in Figure 13A) the five annotated metabolites are ranked according to the normalized amount difference between DAT-DS (Diff). Negative or positive differences means that the metabolites are either more accumulated in DS or in DAT, respectively. Annotations names were obtained from Masstrix queries on the *Vitis vinifera* database (with 1 ppm error), which assigned theoretical masses (ion and neutral) associated to the corresponding raw formula and predicted structure (MSCC); Table S3: Identified Canes metabolites from treated and non-treated vines (DAT-c) versus (DS-c). From 113 significant *m*/*z* (*P*: ANOVA *p* value) discriminating the two conditions DAT and DS canes (see Volcano in Figure 13B) the 32 annotated metabolites are ranked according to the normalized amount difference between DAT-DS (Diff). Negative or positive differences means that the metabolites are either more accumulated in DS or in DAT, respectively. Annotations names were obtained from Masstrix queries on the *Vitis vinifera* database (with 1 ppm error), which assigned theoretical masses (ion and neutral) associated to the corresponding raw formula and predicted structure (MSCC); Table S4: List of fungal species detected in the woody subsamples of vines belonging to the three different conditions analyzed in 2019. Table S5: Grape juices enological parameters obtained from the analysis of two distinct grape juices from five distinct vine trunks, for each of the three vine conditions (only four vine trunks for the H condition).
