# Supplementary material for: Assessment of a New Copper-Based Formulation to Control Esca Disease in Field and Study of Its Impact on the Vine Microbiome, Vine Physiology and Enological Parameters of the Juice
Source: J Fungi (Basel). 2022 Jan 31;8(2):151. doi: 10.3390/jof8020151 (PMC8879249; doi:10.3390/jof8020151)
Supplement: Supplementary file 1 [file jof-08-00151-s001.zip › Supplementary Tables.pdf]

SUPPLEMENTARY Tables

Supplementary Table S1. List of the genes targeted in the transcriptomic study.

Function	Gene	Primer Sequences	GenBank or TC TIGR* Accession Number
Reference genes	*EF1* (EF1-α elongation factor)	5′-GAACTGGGTGCTTGATAGGC-3′<br>5′-AACCAAAATATCCGGAGTAAAAGA-3′	GU585871
	60SRP (60S ribosomal protein L18)	5′-ATCTACCTCAAGCTCCTAGTC-3′<br>5′-CAATCTTGTCCTCCTTTCCT-3′	XM_002270599
	39SRP (39S ribosomal protein L41*)*	5′-AACCAAAATATCCGGAGTAAAAGA-3′<br>5′-GACTGACTTCAAGCTTAAACC-3′	XM_002285709
Phenylpropanoid metabolism	*PAL* (Phenylalanine ammonia lyase)	5′-TCCTCCCGGAAAACAGCTG-3′<br>5′-TCCTCCAAATGCCTCAAATCA-3′	X75967
	STS (Stilbene synthase)	5′-AGGAAGCAGCATTGAAGGCTC-3′<br>5′-TGCACCAGGCATTTCTACACC-3′	FJ851185
Defense protein	*CHIT4C* (Chitinase class V)	5′-TCGAATGGGATGGTGGAAA -3′<br>5′-TCCCCTGTCGAAACACCAAG -3′	NM_001281244
	GLUC (β-1,3 glucanase)	5′-TCAATGGCTGCAATGGTGC-3′<br>5′-CGGTCGATGTTGCGAGATTTA-3′	DQ267748
	PR1<br>(pathogenesis-related protein 1)	5′- GGAGTCCATTAGCACTCCTTTG -3′<br>5′- CATAATTCTGGGCGTAGGCAG -3′	XM_002273752
	PR10<br>(pathogenesis-related protein 10)	5′- CGTTAAGGGCGGCAAAGAG -3′<br>5′- GCATCAGGGTGTGCCAAGA -3′	DQ396809
Detoxification and Stress tolerance	*GST1* (Glutathione s-transferase 1)	5′- TGCATGGAGGAGGAGTTCGT -3′<br>5′- CAAGGCTATATCCCCATTTTCTTC -3′	NM_001281248
Photosynthesis	*PsbP1 (oxygen-evolving enhancer PsbP subunit of photosystem I)*	5′-TGTCCCCAGCCTGTACCTTG-3′<br>5′-GCTGACGGAGATGAAGGTGG-3′	XM_002283012.4
	RbcL (RuBisCo large sub-unit)	5′-AATTTTTCCTCCACGGCGATA -3′<br>5′-ATCTGCGCCCGCCTTTATA -3′	TC57584
Arsenite recovery genes	*MSR* (Peptide methionine sulfoxide reductase))	5′-GCATTTGGGCGTGTAGATGG-3′<br>5′-GCTTCAGTGTGGCCTGTTCT -3′	XM_010662104.2
	WRKY (transcription factors)	5′-GGGCAGAAGGACATTCTTGGA -3′<br>5′-AGGGATCTTCATCCGAACGC -3′	XM_002272684.3
	HYD2 (ABA 8' hydroxylase 2)	5′-GAAGCTTGTTCTCCGAGCCT -3′<br>5′-TATTGATCGACCGGCCTTCC -3′	NM_001281052.1

* see http://www.jcvi.org/cms/research/projects/tdb/overview/

Supplementary Table S2: Identified asymptomatic Leaves metabolites from treated and non-treated vines (DAT-la) versus (DS-la). From 16 significant *m/z* (*P*: ANOVA p value) discriminating the two conditions DAT and DS asymptomatic leaves (*see* Volcano in Figure 13A) the 5 annotated metabolites are ranked according to the normalized amount difference between DAT-DS (Diff). Negative or positive differences means that the metabolites are either more accumulated in DS or in DAT, respectively. Annotations names were obtained from Masstrix queries on *Vitis vinifera* database (with 1 ppm error), which assigned theoretical masses (ion and neutral) associated to the corresponding raw formula and predicted structure (MSCC).

ID	*P*	Diff	Th Mass (Ion)	Structure	Formula	Th Mass (Neutral)	Error (ppm)	Names
1581	0.000272	-4.60	338.088130	Ph	$C_{15}H_{17}NO_8$	339.095420	0.441	6-Hydroxy-5-methoxyindole glucuronide (see KEGG C03033); (2S,3S,4S,5R,6S)-3,4,5-trihydroxy-6-[(5-methoxy-1H-indol-6-yl)oxy]oxane-2-carboxylic acid [acetal]
3316	0.004486	-4.16	413.218150	Lp	$C_{21}H_{34}O_8$	414.225589	0,535	methyl 5-hydroperoxy-6,8,9,11-bisepidioxy-12,14-eicosadienoate [Hydroperoxy fatty acids [FA0104]]
3963	0.002901	-3.60	437.436393	Lp	$C_{29}H_{58}O_2$	438.443680	-0,001	Nonacosanoic acid; nonacosanoic acid [carboxylic acid]
189	0.000046	-1.18	219.051039	NM	$C_8H_{12}O_7$	220.058305	0,012	(R)-(Homo) 2-citrate; 1-Hydroxypentane-1,2,5-tricarboxylate; (-) threo-iso(homo) 2 citrate; 3-Hydroxy-3-carboxymethyl-adipic acid
2193	0.000682	+0.95	365.064435	NM	$C_{13}H_{19}O_{10}P$	366.071588	0,013	Salicin 6-phosphate; Salicin-6P

Supplementary Table S3 : Identified Canes metabolites from treated and non-treated vines (DAT-c) versus (DS-c). From 113 significant *m*/*z* (*P*: ANOVA p value) discriminating the two conditions DAT and DS canes (*see* Volcano in Figure 13B) the 32 annotated metabolites are ranked according to the normalized amount difference between DAT-DS (Diff). Negative or positive differences means that the metabolites are either more accumulated in DS or in DAT, respectively. Annotations names were obtained from Masstrix queries on *Vitis vinifera* database (with 1 ppm error), which assigned theoretical masses (ion and neutral) associated to the corresponding raw formula and predicted structure (MSCC).

ID	*P*	Diff	Th Mass (Ion)	Structure	Formula	Th Mass (Neutral)	Error (ppm)	Names
1913	0.010626	-4.21	353.103146	Ph	$C_{20}H_{18}O_6$	354.110340	0.006	Cyclokievitone
212	0.009158	-3.62	223.13398	Lp	$C_{13}H_{20}O_3$	224.141245	0.004	Vomifoliol; (6S,9R)-6-hydroxy-3-oxo-�-ionol
9268	0.007012	-3.55	629.172184	Ph	$C_{27}H_{34}O_{17}$	630.179591	-0.012	Leucodelphinidin 3-O-(�-D-glucopyranosyl-(1->4)-�-L-rhamnopyranoside) [Flavans, Flavanols and Leucoanthocyanidins [PK1202]]
4910	1.18E-07	-3.40	471.384416	Lp	$C_{31}H_{52}O_3$	472.391645	-0.000	Soyasapogenol D
9149	0.027266	-3.39	623.161585	Ph	$C_{28}H_{32}O_{16}$	624.168680	-0.568	Tectorigenin 7-O-gentiobioside [Isoflavonoids [PK1205]]
8162	0.041731	-2.93	575.452665	Lp	$C_{32}H_{64}O_8$	576.460120	0.002	1-(O-�-D-glucopyranosyl)-(1,3R,25R)-hexacosanetriol [Fatty acyl glycosides of mono- and disaccharides [FA1301]]
8734	0.015264	-2.76	603.484088	Lp	$C_{34}H_{68}O_8$	604.491420	0.002	1-(O-�-D-glucopyranosyl)-(1,3R,27R)-octaconsanetriol [Fatty acyl glycosides of mono- and disaccharides [FA1301]]
2470	0.000113	-2.75	377.160518	Ph	$C_{20}H_{26}O_7$	378.167631	-0.587	Chaparrinone
6583	0.022281	-2.72	517.389808	Lp	$C_{32}H_{54}O_5$	518.397125	0.001	7�-acetoxy-gorgostan-3�,5�,6�-triol [Gorgosterols and derivatives[ST0106]]
3850	0.034607	-2.45	433.114036	Ph	$C_{21}H_{22}O_{10}$	434.121138	-0.365	Naringenin 7-O-�-D-glucoside; Prunin
168	0.003001	-2.39	215.032805	Cb	$C_5H_{13}O_7P$	216.040068	0.832	2-C-Methyl-D-erythritol 4-phosphate
398	0.017330	-2.29	252.051365	Ph	$C_{11}H_{11}NO_6$	253.058639	0.009	N-Pyruvoyl-5-methoxy-3-hydroxyanthranilate; N-Pyruvoyl-5-methoxy-3-hydroxyanthranilic acid
4275	0.010541	-2.17	449.108874	Ph	$C_{21}H_{22}O_{11}$	450.116228	0.037	Neoastilbin; (2S,3S)-Taxifolin 3-rhamnoside
3963	0.005772	-2.03	437.436392	Lp	$C_{29}H_{58}O_2$	438.443680	-0.001	Nonacosanoic acid; nonacosanoic acid [carboxylicacid]
9827	0.043267	-2.00	659.416826	Lp	$C_{38}H_{60}O_9$	660.424359	0.949	12-O-Palmitoyl-16-hydroxyphorbol 13-acetate; Crotonfactor F1
3595	0.019732	-1.86	423.420748	Lp	$C_{28}H_{56}O_2$	424.428030	-0.001	Octacosanoic acid; octacosanoic acid [carboxylicacid]
2193	0.019086	-1.70	365.064434	Cb	$C_{13}H_{19}O_{10}P$	366.071588	0.013	Salicin 6-phosphate; Salicin-6P
4370	0.021845	-1.65	451.452007	Lp	$C_{30}H_{60}O_2$	452.459330	-0.001	Melissic acid; triacontanoic acid [Straight chain fatty acids [FA0101]]
3437	0.041210	-1.65	419.098345	Ph	$C_{20}H_{20}O_{10}$	420.105480	-0.396	5,3',5'-Trihydroxy-3,6,7,8,4'-pentamethoxyflavone [Flavones and Flavonols [PK1211]]
2711	0.035887	-1.38	387.129652	Lp	$C_{17}H_{24}O_{10}$	388.136901	-0.117	Secologanin; (-)-Secologanin
1466	0.026006	-1.25	333.061614	Ph	$C_{16}H_{14}O_8$	334.068821	-0.137	6-Methoxytaxifolin
8192	0.041414	-1.24	577.135010	Ph	$C_{30}H_{26}O_{12}$	578.142443	0.029	Epicatechin-(4�->8)-ent-epicatechin
1341	0.020785	-1.21	327.10854	Ph	$C_{15}H_{20}O_8$	328.115820	0.008	Anisatin
8092	0.034853	-1.01	571.327453	Lp	$C_{33}H_{48}O_8$	572.334750	-0.294	6,8a-Seco-6,8a-deoxy-5-oxoavermectin''2b''a glycone
6493	0.035506	-0.93	515.192200	Pp	$C_{14}H_{33}N_{10}O_7PS$	516.199536	0.651	Phaseolotoxin
1699	0.009584	-0.73	343.124559	Cb	$C_{12}H_{24}O_{11}$	344.131972	0.323	Melibiitol; 6-O-�-D-Galactosyl-D-glucitol
476	0.041925	-0.67	263.128880	Ph	$C_{15}H_{20}O_4$	264.136160	0.005	Abscisate; Abscisic acid; (+)-Abscisic acid
1291	0.025648	-0.45	325.114000	Cb	$C_{12}H_{21}O_{10}R$	326.121300	0.010	R replaced by H in �-L-Fucosyl-1,2-�-D-galactoside; Glycoprotein �-L-fucosyl-(1,2)-D-galactose; Glycolipid �-L-fucosyl-1,2-�-D-galactose; �-L-Fucosyl-(1->2)-D-galactosyl-R
2044	0.034599	-0.45	359.098344	NM	$C_{15}H_{20}O_{10}$	360.105488	-0.441	3-Methoxy-4-hydroxyphenylglycol glucuronide (see KEGG C03033); (2S,3S,4S,5R,6R)-3,4,5-trihydroxy-6-[2-hydroxy-2-(4-hydroxy-3-methoxy-phenyl) ethoxy]oxane-2-carboxylic acid [acetal]

4909	1.26E-05	+4.75	471.38413	Lp	$C_{31}H_{52}O_3$	472.391300	-0.732	Soyasapogenol D
5268	0.034407	+0.89	481.135032	Ph	$C_{22}H_{26}O_{12}$	482.142070	-0.739	Catalposide
5975	0.012284	+0.26	501.145887	Cb	$C_{18}H_{30}O_{16}$	502.153390	0.011	[illegible]-L-Rhamnopyranosyl-(1->2)-[illegible]-D-galactopyranosyl-(1->2)-[illegible]-D-glucuronopyranoside; [illegible]-Fabatriose

Supplementary Table S4. List of fungal species detected in the woody subsamples of vines belonging to the three different conditions analyzed in 2019. *In bold the genera/species associated to GTDs*.

	DAT				DS				H			
Fungal species	c	hw	iw	wr	c	hw	iw	wr	c	hw	iw	wr
Acremonium alcalophilum											Detected	
Acremonium alternatum		Detected	Detected					Detected			Detected	
Acremonium blochii		Detected	Detected									
Acremonium furcatum		Detected	Detected					Detected		Detected		
Acremonium sp.			Detected	Detected				Detected				
Acremonium spinosum				Detected								
Acrostalagmus luteoalbus											Detected	Detected
Agaricus arvensis				Detected							Detected	
Agrocybe praecox								Detected		Detected	Detected	
Ajellomyces crescens	Detected											
Alternaria alternata	Detected	Detected	Detected		Detected	Detected			Detected	Detected	Detected	
Alternaria atra						Detected				Detected		
Alternaria soliaridae										Detected		
Amycosphaerella africana								Detected				
Antarctomyces psychrotrophicus					Detected	Detected		Detected		Detected	Detected	
Arachnomyces kanei							Detected					
Archaeospora schenckii					Detected							
Armillaria mellea	Detected											
Arthothelium spectabile				Detected								
Articulospora sp.	Detected			Detected	Detected			Detected	Detected		Detected	
Ascochyta hordei				Detected				Detected				
Ascochyta manawaorae					Detected							
Aspergillus amstelodami	Detected	Detected	Detected	Detected	Detected	Detected	Detected	Detected	Detected	Detected	Detected	
Aspergillus felis					Detected							
Aspergillus halophilicus						Detected						
Aspergillus japonicus					Detected							
Aspergillus penicillioides	Detected			Detected				Detected		Detected	Detected	
Aspergillus piperis	Detected	Detected	Detected		Detected	Detected	Detected		Detected			
Aspergillus sydowii	Detected	Detected	Detected	Detected	Detected	Detected	Detected	Detected	Detected	Detected	Detected	Detected
Aspergillus wentii					Detected							Detected
Astraeus hygrometricus		Detected										
Athelia pyriformis				Detected								
Aureobasidium pullulans	Detected	Detected	Detected	Detected	Detected	Detected	Detected	Detected	Detected	Detected	Detected	Detected
Auxarthron alboluteum			Detected									
Auxarthron umbrinum				Detected								
Bacidia chloroticula		Detected	Detected	Detected		Detected		Detected		Detected	Detected	
Bacidia neosquamulosa			Detected	Detected				Detected			Detected	Detected
Bacidina adastra		Detected	Detected	Detected		Detected		Detected		Detected	Detected	Detected
Baeospora myosura	Detected							Detected				
Basidioascus undulatus					Detected							
Beauveria pseudobassiana												Detected
Bipolaris eleusines	Detected											
Bisporella subpallida				Detected								
Boeremia exigua	Detected	Detected	Detected	Detected	Detected			Detected			Detected	Detected
Botryosphaeria dothidea	Detected											
Botryotinia fuckeliana	Detected	Detected	Detected	Detected	Detected		Detected	Detected	Detected	Detected	Detected	Detected
Brachyphoris oviparasitica					Detected							
Bradymyces alpinus										Detected		
Brettanomyces bruxellensis								Detected				
Brevicellicium olivascens				Detected								
Cadophora luteo-olivacea		Detected	Detected	Detected		Detected		Detected			Detected	Detected
Cadophora melinii			Detected									
Cadophora sp.			Detected									
Calcarisporiella thermophila		Detected	Detected		Detected	Detected	Detected	Detected	Detected	Detected	Detected	
Calocera cornea				Detected								
Caloplaca obscurella								Detected				
Calycina claroflava				Detected				Detected		Detected		Detected
Camarosporium sp.								Detected				
Candida orthopsilosis										Detected		

Candida parapsilosis	Detected								Detected			
Candida subhashii		Detected				Detected						
Candida zeylanoides			Detected									
Capnobotryella sp.		Detected						Detected				
Capronia pilosella			Detected	Detected		Detected		Detected			Detected	Detected
Capronia semi-immersa			Detected	Detected				Detected				Detected
Capronia sp.	Detected		Detected	Detected	Detected	Detected		Detected	Detected	Detected		
Cephalosporium serrae		Detected		Detected	Detected	Detected			Detected			
Ceratocystis sp.			Detected								Detected	
Cercospora apii		Detected										
Chaetomium iranianum	Detected		Detected						Detected		Detected	
Chaetomium sp.	Detected				Detected					Detected		
Chaetosphaeria myriocarpa			Detected									
Chaetosphaeria sp.			Detected									
Chalastospora ellipsoidea	Detected			Detected	Detected			Detected	Detected			Detected
Chalastospora obclavata	Detected			Detected	Detected			Detected		Detected	Detected	Detected
Chrysosporium lobatum			Detected								Detected	
Chrysosporium pilosum	Detected		Detected			Detected			Detected	Detected	Detected	
Chrysosporium sp.	Detected				Detected			Detected			Detected	
Cistella sp.			Detected									
Cladophialophora chaetospira										Detected		
Cladophialophora sp.			Detected	Detected	Detected	Detected	Detected	Detected	Detected		Detected	
Cladosporium halotolerans						Detected				Detected	Detected	
Cladosporium herbarum	Detected	Detected	Detected	Detected	Detected	Detected	Detected	Detected	Detected	Detected	Detected	Detected
Cladosporium ramotenellum	Detected	Detected	Detected		Detected	Detected		Detected		Detected		Detected
Cladosporium sphaerospermum		Detected	Detected	Detected		Detected				Detected	Detected	
Clavaria californica						Detected						
Claviceps sp.	Detected	Detected	Detected	Detected				Detected		Detected	Detected	Detected
Clitocybe rivulosa		Detected										
Clitopilus hobsonii											Detected	
Clonostachys rosea			Detected							Detected		
Cochliobolus geniculatus						Detected						
Coniolariella hispanica		Detected		Detected		Detected		Detected				
Coniosporium sp.								Detected				
Coniothyrium sidae		Detected	Detected		Detected			Detected	Detected		Detected	
Conocybe sp.				Detected								
Coprinellus micaceus	Detected											
Coprinellus radians	Detected									Detected		
Coprinellus sabulicola	Detected		Detected		Detected				Detected			
Coprinellus sp.										Detected		
Cortinarius sp.	Detected	Detected			Detected		Detected	Detected	Detected	Detected	Detected	
Corynespora smithii		Detected		Detected		Detected		Detected		Detected	Detected	Detected
Cosmospora sp.		Detected	Detected	Detected		Detected		Detected		Detected	Detected	Detected
Crocicreas sp.				Detected								
Cryptococcus adeliensis	Detected				Detected		Detected	Detected		Detected	Detected	
Cryptococcus aerius	Detected	Detected	Detected	Detected	Detected	Detected	Detected	Detected	Detected		Detected	
Cryptococcus bhutanensis	Detected	Detected	Detected		Detected				Detected			
Cryptococcus cerealis									Detected			
Cryptococcus chernovii	Detected	Detected		Detected	Detected	Detected		Detected	Detected	Detected	Detected	Detected
Cryptococcus diffluens	Detected	Detected	Detected		Detected	Detected		Detected		Detected		
Cryptococcus dimennae	Detected		Detected	Detected	Detected			Detected				
Cryptococcus friedmannii		Detected										
Cryptococcus heimaeyensis	Detected											
Cryptococcus laurentii		Detected	Detected	Detected	Detected			Detected		Detected		
Cryptococcus magnus	Detected	Detected	Detected		Detected			Detected	Detected	Detected		
Cryptococcus oeirensis	Detected				Detected							
Cryptococcus phenolicus		Detected	Detected	Detected	Detected	Detected			Detected	Detected	Detected	Detected
Cryptococcus podzolicus	Detected					Detected			Detected			
Cryptococcus saitoi												Detected
Cryptococcus sp.	Detected	Detected	Detected	Detected	Detected	Detected		Detected	Detected	Detected	Detected	Detected
Cryptococcus terreus	Detected	Detected	Detected		Detected	Detected			Detected	Detected		
Cryptococcus terricola	Detected					Detected						
Cryptococcus victoriae	Detected	Detected	Detected	Detected	Detected		Detected	Detected		Detected		
Cryptococcus wieringae										Detected		
Cryptodiscus rhopaloides				Detected				Detected				Detected
Cryptosporiopsis actinidiae				Detected				Detected			Detected	
Curreya austroafricana		Detected	Detected	Detected		Detected		Detected		Detected		Detected
Curvibasidium cygneicollum								Detected				

Curvularia gladioli						Detected						
Cylindrotrichum gorii			Detected					Detected				
Cystobasidium slooffiae		Detected										
Cystodendron sp.				Detected								
Cystofilobasidium capitatum				Detected				Detected			Detected	
Cystofilobasidium macerans					Detected			Detected				Detected
Dactylonectria alcacerensis	Detected	Detected	Detected	Detected	Detected		Detected					
Dactylonectria pauciseptata	Detected											
Dendryphion europaeum												Detected
Devriesia sp.		Detected	Detected	Detected		Detected		Detected		Detected	Detected	Detected
Didymella sp.				Detected								Detected
Didymosphaeria futilis	Detected	Detected	Detected	Detected	Detected	Detected	Detected	Detected		Detected	Detected	Detected
Didymosphaeria sp.			Detected				Detected					
Diplodia seriata		Detected	Detected	Detected		Detected		Detected		Detected	Detected	Detected
Entoleuca sp.		Detected										
Entoloma platyphylloides	Detected											
Epicoccum nigrum	Detected	Detected	Detected	Detected	Detected	Detected		Detected	Detected	Detected	Detected	Detected
Erysiphe arcuata					Detected							
Erysiphe heraclei					Detected							
Erythrobasidium hasegawianum					Detected					Detected	Detected	
Eutypa lata		Detected	Detected	Detected			Detected	Detected		Detected	Detected	Detected
Eutypa leptoplaca					Detected							
Exophiala brunnea				Detected								
Exophiala equina											Detected	
Exophiala moniliae				Detected				Detected				
Exophiala opportunistica				Detected				Detected				
Exophiala sp.		Detected	Detected	Detected				Detected			Detected	Detected
Exophiala xenobiotica								Detected				
Fellomyces horovitziae				Detected								
Fellomyces mexicanus		Detected				Detected		Detected			Detected	
Fellomyces sp.				Detected				Detected				Detected
Fellomyces thailandicus		Detected		Detected				Detected		Detected	Detected	Detected
Fibulobasidium inconspicuum							Detected					
Filobasidium floriforme	Detected											
Flagelloscypha minutissima						Detected						
Flagelloscypha sp.										Detected		
Flavoplaca sp.								Detected				
Fomitiporia aethiopica	Detected	Detected	Detected	Detected	Detected	Detected	Detected	Detected	Detected	Detected	Detected	Detected
Fomitiporia mediterranea	Detected	Detected	Detected	Detected	Detected	Detected	Detected	Detected	Detected	Detected	Detected	Detected
Fomitiporia polymorpha		Detected	Detected	Detected		Detected	Detected	Detected		Detected		Detected
Fusarium acuminatum		Detected		Detected				Detected				Detected
Fusarium beomiforme	Detected						Detected					
Fusarium boothii				Detected								
Fusarium pseudensiforme	Detected	Detected	Detected	Detected	Detected	Detected	Detected	Detected	Detected		Detected	Detected
Fusarium sp.					Detected							
Fusidium griseum		Detected		Detected				Detected		Detected	Detected	Detected
Geopora cervina					Detected							
Geopyxis sp.		Detected		Detected					Detected			
Geotrichum klebahnii					Detected							
Guehomyces pullulans					Detected			Detected	Detected			
Gymnascella aurantiaca			Detected					Detected				
Gymnopus dryophilus	Detected											
Gymnostellatospora frigida						Detected				Detected	Detected	
Hannaella oryzae	Detected				Detected	Detected					Detected	
Hannaella surugaensis		Detected	Detected	Detected		Detected						Detected
Hannaella zeae	Detected											
Hanseniaspora thailandica			Detected									
Hanseniaspora uvarum	Detected	Detected	Detected		Detected		Detected			Detected	Detected	
Helgardia anguioides	Detected							Detected				
Helicoma sp.		Detected										
Hemimycena cucullata		Detected	Detected	Detected				Detected			Detected	Detected
Herpotrichia parasitica				Detected				Detected				Detected
Heteroacanthella acanthophysa								Detected				
Holtermannia corniformis					Detected							
Humicola grisea	Detected				Detected						Detected	
Hydropisphaera sp.			Detected		Detected			Detected				
Hyphodermella corrugata								Detected	Detected			
Hyphodontia radula					Detected				Detected			

Hypholoma fasciculare				Detected								
Ilyonectria liriodendri		Detected	Detected							Detected	Detected	Detected
Kavinia alboviridis											Detected	
Lachancea thermotolerans				Detected								
Lachnum cerinum				Detected								
Lachnum sp.		Detected										
Lalaria carpini										Detected		
Lasiobelonium nidulum								Detected				
Lecania cyrtella	Detected	Detected		Detected		Detected		Detected				Detected
Lecania cyrtellina				Detected				Detected				Detected
Lecania proteiformis				Detected							Detected	
Lecanicillium muscarium			Detected	Detected		Detected		Detected			Detected	Detected
Lecanicillium saksenae						Detected						
Lecanicillium sp.		Detected										
Lecanora albescens				Detected				Detected		Detected	Detected	
Lecanora dispersa											Detected	
Lecanora hagenii			Detected	Detected		Detected		Detected		Detected		
Lecythophora sp.			Detected	Detected		Detected		Detected			Detected	Detected
Leohumicola minima			Detected									
Lepiota brunneoincarnata	Detected		Detected		Detected	Detected		Detected	Detected	Detected		
Lepiota echinella									Detected			
Lepista inversa				Detected								
Leptosphaeria sp.			Detected									Detected
Leptosphaerulina australis				Detected					Detected			Detected
Lewia infectoria	Detected	Detected		Detected	Detected	Detected	Detected	Detected	Detected	Detected	Detected	Detected
Limonomyces roseipellis	Detected			Detected	Detected							
Lipomyces chichibuensis					Detected			Detected	Detected		Detected	
Lophiostoma sp.		Detected	Detected			Detected				Detected	Detected	
Malassezia globosa		Detected								Detected		Detected
Malassezia restricta		Detected		Detected			Detected			Detected	Detected	
Malassezia sp.	Detected	Detected	Detected	Detected		Detected	Detected			Detected		Detected
Massarina sp.	Detected	Detected	Detected	Detected	Detected	Detected		Detected		Detected		Detected
Metarhizium anisopliae								Detected				
Metschnikowia chrysoperlae	Detected											
Metschnikowia pulcherrima	Detected	Detected				Detected						
Metschnikowia sp.	Detected		Detected	Detected								
Meyerozyma guilliermondii	Detected	Detected	Detected		Detected	Detected	Detected					
Microascus sp.	Detected	Detected	Detected	Detected	Detected	Detected	Detected	Detected	Detected	Detected	Detected	
Microdiplodia sp.					Detected	Detected	Detected	Detected	Detected	Detected	Detected	
Monographella cucumerina						Detected						
Monographella nivalis								Detected				
Morchella semilibera										Detected		Detected
Mortierella camargensis					Detected		Detected					
Mortierella clonocystis					Detected						Detected	
Mortierella elongata	Detected	Detected	Detected	Detected	Detected							Detected
Mortierella rishikesha	Detected								Detected			
Mortierella sp.	Detected	Detected	Detected	Detected	Detected	Detected	Detected	Detected	Detected	Detected	Detected	Detected
Mortierella stylospora			Detected	Detected		Detected					Detected	
Mucor brunneogriseus								Detected				
Mycena capillaripes										Detected	Detected	
Mycena cicognanii		Detected		Detected								Detected
Mycena sp.	Detected										Detected	
Mycena terena		Detected	Detected									
Mycenella bryophila		Detected				Detected		Detected				Detected
Mycenella trachyspora			Detected	Detected				Detected				Detected
Mycoleptodiscus sp.			Detected		Detected							
Nectria cinnabarina											Detected	
Nectria ramulariae											Detected	
Nematoctonus leiosporus								Detected				Detected
Neoascochyta exitialis	Detected											
Neopeckia coulteri		Detected	Detected		Detected			Detected		Detected	Detected	
Neophaeosphaeria sp.			Detected									
Neosetophoma sp.								Detected			Detected	
Neurospora terricola												Detected
Nidulariopsis iowensis		Detected		Detected				Detected		Detected		
Nigroporus vinosus												Detected
Ochroconis sp.		Detected										
Oidiodendron griseum	Detected							Detected				

Olpidium brassicae		Detected	Detected	Detected		Detected		Detected		Detected	Detected	
Paecilomyces marquandii	Detected		Detected									
Paraconiothyrium fuckelii										Detected		
Paraconiothyrium variabile												Detected
Paraphoma chrysanthemicola									Detected			
Paraphoma sp.				Detected	Detected	Detected		Detected		Detected		
Parasola lilatincta											Detected	
Parastagonospora avenae								Detected				
Penicillium brevicompactum		Detected	Detected		Detected			Detected		Detected	Detected	Detected
Penicillium brocae					Detected							
Penicillium cairnsense		Detected								Detected		
Penicillium citrinum					Detected							
Penicillium corylophilum		Detected										
Penicillium cyclopium										Detected		
Penicillium expansum		Detected			Detected				Detected			
Penicillium lapidosum	Detected	Detected	Detected	Detected	Detected	Detected	Detected	Detected	Detected	Detected		Detected
Penicillium levitum	Detected	Detected			Detected	Detected		Detected				
Penicillium megasporum											Detected	
Penicillium menonorum	Detected	Detected	Detected	Detected	Detected			Detected	Detected	Detected	Detected	
Penicillium monsgalena		Detected										
Penicillium nodositatum	Detected		Detected	Detected	Detected				Detected	Detected	Detected	Detected
Penicillium ochrochloron		Detected						Detected	Detected			
Penicillium parvum	Detected		Detected		Detected	Detected			Detected	Detected		
Penicillium purpurogenum										Detected		
Penicillium quercetorum									Detected			
Penicillium roseopurpureum		Detected	Detected	Detected	Detected	Detected	Detected		Detected		Detected	
Penicillium sizovae				Detected		Detected				Detected		Detected
Penicillium sp.	Detected	Detected	Detected	Detected	Detected	Detected		Detected	Detected	Detected	Detected	
Penicillium swiecickii		Detected			Detected					Detected		
Penicillium terrigenum	Detected				Detected				Detected			
Penicillium tularense							Detected					
Penicillium westlingii		Detected	Detected	Detected		Detected	Detected	Detected		Detected	Detected	Detected
Perenniporia fraxinea					Detected							
Phaeoacremonium fraxinopennsylvanicum		Detected	Detected			Detected	Detected	Detected		Detected	Detected	Detected
Phaeoacremonium iranianum		Detected	Detected	Detected		Detected	Detected	Detected		Detected	Detected	
Phaeoacremonium viticola		Detected									Detected	
Phaeococcomyces sp.			Detected					Detected		Detected		
Phaeomoniella chlamydospora	Detected	Detected	Detected	Detected	Detected	Detected	Detected	Detected	Detected	Detected	Detected	Detected
Phaeosphaeria sp.	Detected			Detected	Detected			Detected	Detected			Detected
Phakopsora pachyrhizi					Detected							
Phallus rugulosus											Detected	
Phialemoniopsis ocularis			Detected								Detected	
Phialophora livistonae								Detected				
Phlebia uda				Detected					Detected	Detected	Detected	
Phoma sp.	Detected			Detected	Detected				Detected	Detected		
Physcia leptalea		Detected		Detected				Detected			Detected	Detected
Pichia kluyveri	Detected											
Pichia membranifaciens	Detected											
Pleospora herbarum	Detected		Detected	Detected	Detected			Detected	Detected			Detected
Pochonia bulbillosa		Detected			Detected	Detected		Detected				
Podospora communis				Detected								
Podospora sp.		Detected										
Powellomyces sp.										Detected		
Preussia flanaganii		Detected										
Psathyrella sp.											Detected	
Pseudallescheria fimeti	Detected	Detected	Detected		Detected	Detected		Detected				
Pterula echo											Detected	
Puccinia graminis			Detected									
Pyrenochaeta sp.		Detected		Detected				Detected			Detected	
Pyrenochaetopsis leptospora						Detected		Detected				
Pyrenophora catenaria					Detected	Detected	Detected					
Pyrenophora dictyoides					Detected			Detected				
Pyrenophora graminea					Detected							
Pyrenophora lolii						Detected				Detected		
Rhexocercosporidium panacis		Detected		Detected							Detected	
Rhinocladiella sp.		Detected		Detected				Detected				
Rhodotorula eucalyptica				Detected				Detected				

Rhodotorula laryngis	Detected	Detected		Detected	Detected				Detected			Detected
Roesleria subterranea			Detected	Detected								
Saccharomyces cerevisiae	Detected	Detected	Detected			Detected	Detected			Detected		
Sarocladium sp.		Detected										
Sarocladium strictum			Detected									
Sawadaea bicornis										Detected		
Schizothecium carpinicola								Detected		Detected		Detected
Scleromitrula spiraeicola			Detected									
Sclerostagonospora sp.		Detected	Detected	Detected				Detected		Detected	Detected	Detected
Scopuloides hydnoides											Detected	
Sebacina sp.	Detected	Detected	Detected					Detected				Detected
Serendipita vermifera								Detected				
Setophoma terrestris			Detected									
Solicorynespora sp.				Detected								
Spiromastix princeps			Detected		Detected	Detected		Detected				
Sporobolomyces coprosmae	Detected				Detected					Detected		
Sporobolomyces salicinus											Detected	
Sporobolomyces sp.	Detected						Detected				Detected	
Sporobolomyces symmetricus				Detected				Detected				
Stagonosporopsis dorenboschii	Detected											
Starmerella bombicola					Detected							
Stereum hirsutum					Detected			Detected				
Syzygospora sp.		Detected	Detected	Detected				Detected				
Talaromyces purpureogenus	Detected	Detected				Detected	Detected	Detected				
Taphrina wiesneri										Detected		
Tetracladium marchalianum				Detected				Detected				Detected
Tetracladium sp.				Detected	Detected			Detected	Detected			Detected
Torulaspora delbrueckii	Detected		Detected									
Trametes versicolor			Detected						Detected			
Trechispora sp.					Detected			Detected		Detected		
Trematosphaeria pertusa				Detected			Detected	Detected				Detected
Tremella phaeophysciae				Detected	Detected			Detected			Detected	
Trichosporon moniliiforme											Detected	
Truncatella laurocerasi								Detected			Detected	
Tumularia sp.				Detected				Detected				
Udeniomyces puniceus						Detected		Detected		Detected		
Umbelopsis angularis								Detected				
Uncinula necator		Detected				Detected					Detected	
Volutella ciliata							Detected					
Volutella sp.				Detected				Detected				Detected
Waitea circinata										Detected		
Wallemia sebi		Detected	Detected		Detected		Detected	Detected				
Wickerhamomyces sp.					Detected							
Wojnowicia viburni												Detected
Xanthoria coomae				Detected				Detected				
Xenasmatella christiansenii	Detected	Detected		Detected		Detected		Detected	Detected		Detected	
Xylodon sambuci								Detected				
Zignoella pulviscula								Detected				

Supplementary Table S5: Grape juices enological parameters obtained from the analysis of two distinct grape juices from five distinct vine trunks, for each of the three vine conditions (only 4 vine trunks for the H condition).

	pH[a]		α-amino Nitrogen[b] (mg/L)		Ammoniacal Nitrogen[a] (mg/L)		TA[a] (g/L Sulfuric acid)		Sugars[a] (g/L)	
**Sample**	**Average**	**STD**	**Average**	**STD**	**Average**	**STD**	**Average**	**STD**	**Average**	**STD**
DS1	2.74	0.06	90	15	99.55	0.05	8.7	0.7	100	12
DS2	2.91	0.01	84	5	85	3	7.9	0.1	169	4
DS3	2.83	0.03	93	7	86	6	9.1	0.1	133	2
DS4	2.94	0.00	241	17	361	11	11.7	0.4	56	3
DS5	2.81	0.10	55	1	82	3	7.4	0.5	155	14
DAT1	2.85	0.03	61	2	74	2	6.07	0.18	167	6
DAT2	2.83	0.01	79	5	123	7	6.69	0.12	156	3
DAT3	2.87	0.01	78.2	0	93	2	6.6	0.1	171	3
DAT4	2.95	0.01	46	9	45	2	6.96	0.09	145.1	3.0
DAT5	2.82	0.04	81	7	82	6	6.9	0.3	164	9
H1	2.98	0.01	68	4	66.9	0.9	7.6	0.1	176	3
H2	3.06	0.03	210	29	237.1	0.5	6.5	0.0	178	2
H3	2.93	0.01	95	9	88	5	7.54	0.05	175.1	1.8
H5	2.99	0.02	19	3	45	0.4	7.0	0.1	154	5

a. FTIR : Fourier Transformed Infra Red; b. enzymatic kit ; TA :total Acidity ; STD : Standard Deviation
