# Supplementary figures and images for: Assessment of a New Copper-Based Formulation to Control Esca Disease in Field and Study of Its Impact on the Vine Microbiome, Vine Physiology and Enological Parameters of the Juice
Source: J Fungi (Basel). 2022 Jan 31;8(2):151. doi: 10.3390/jof8020151 (PMC8879249; doi:10.3390/jof8020151)

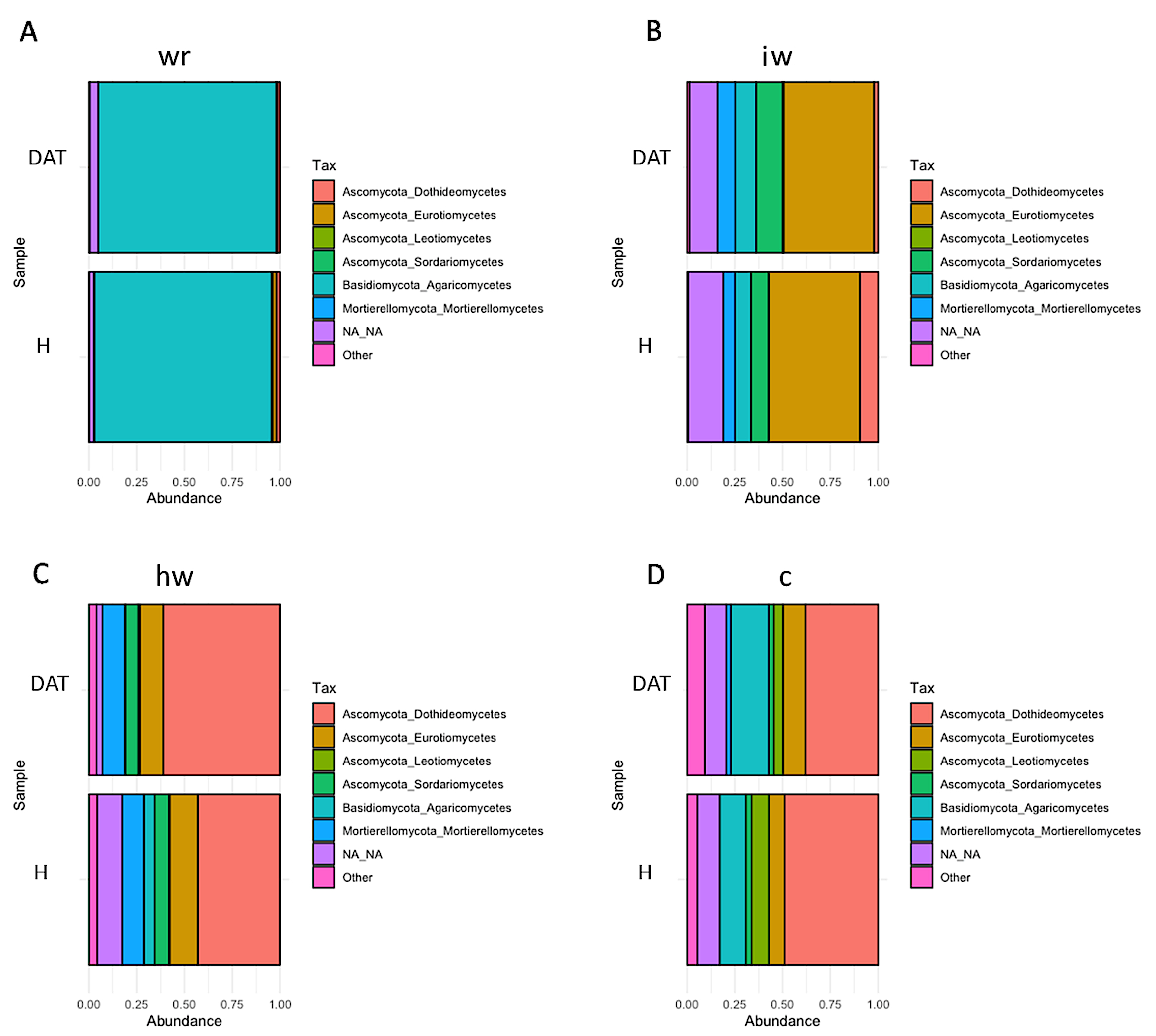

Supplement: Supplementary file 1 [file jof-08-00151-s001.zip › Figure S1.jpg]

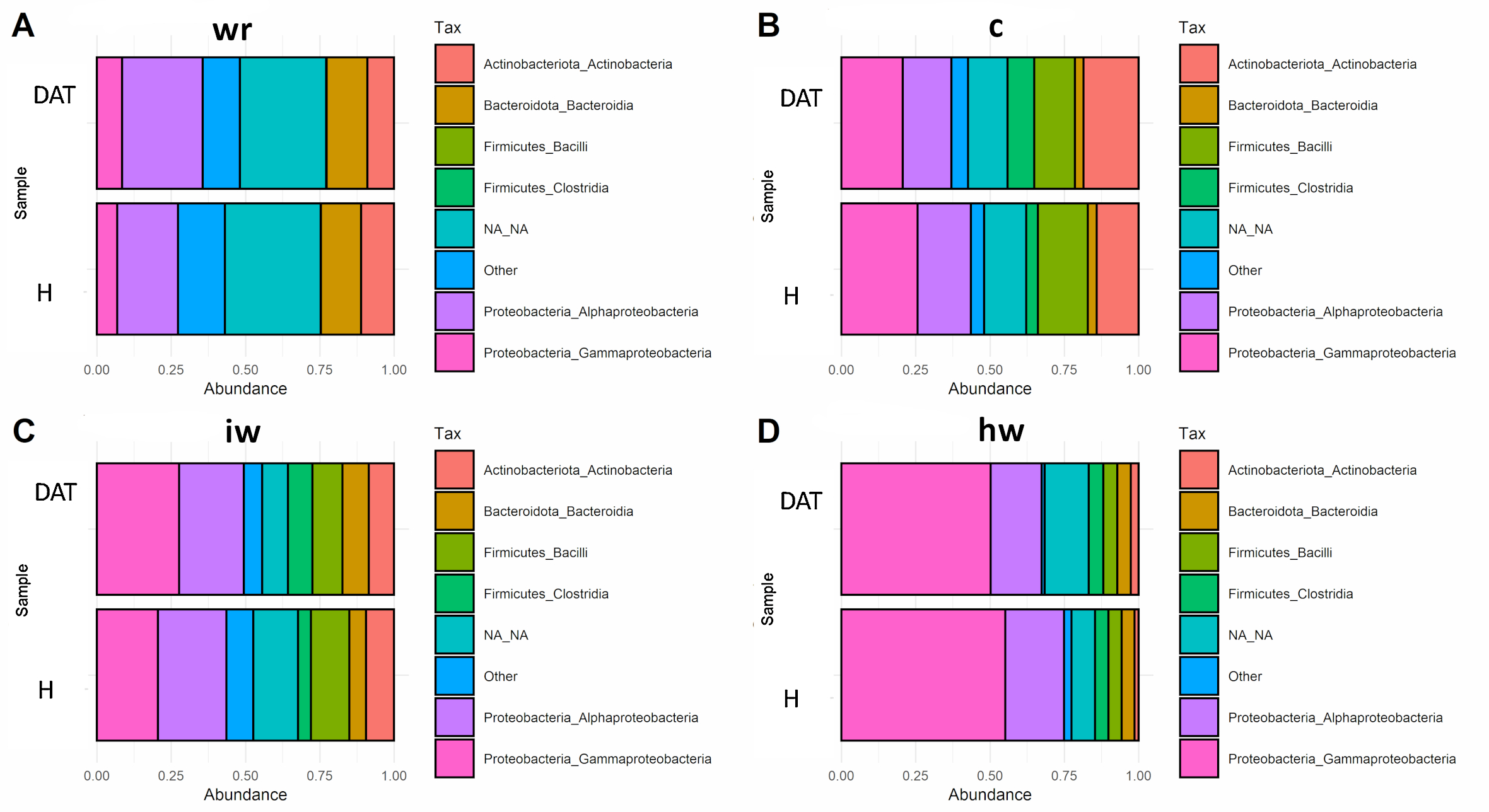

Supplement: Supplementary file 1 [file jof-08-00151-s001.zip › Figure S2.jpg]

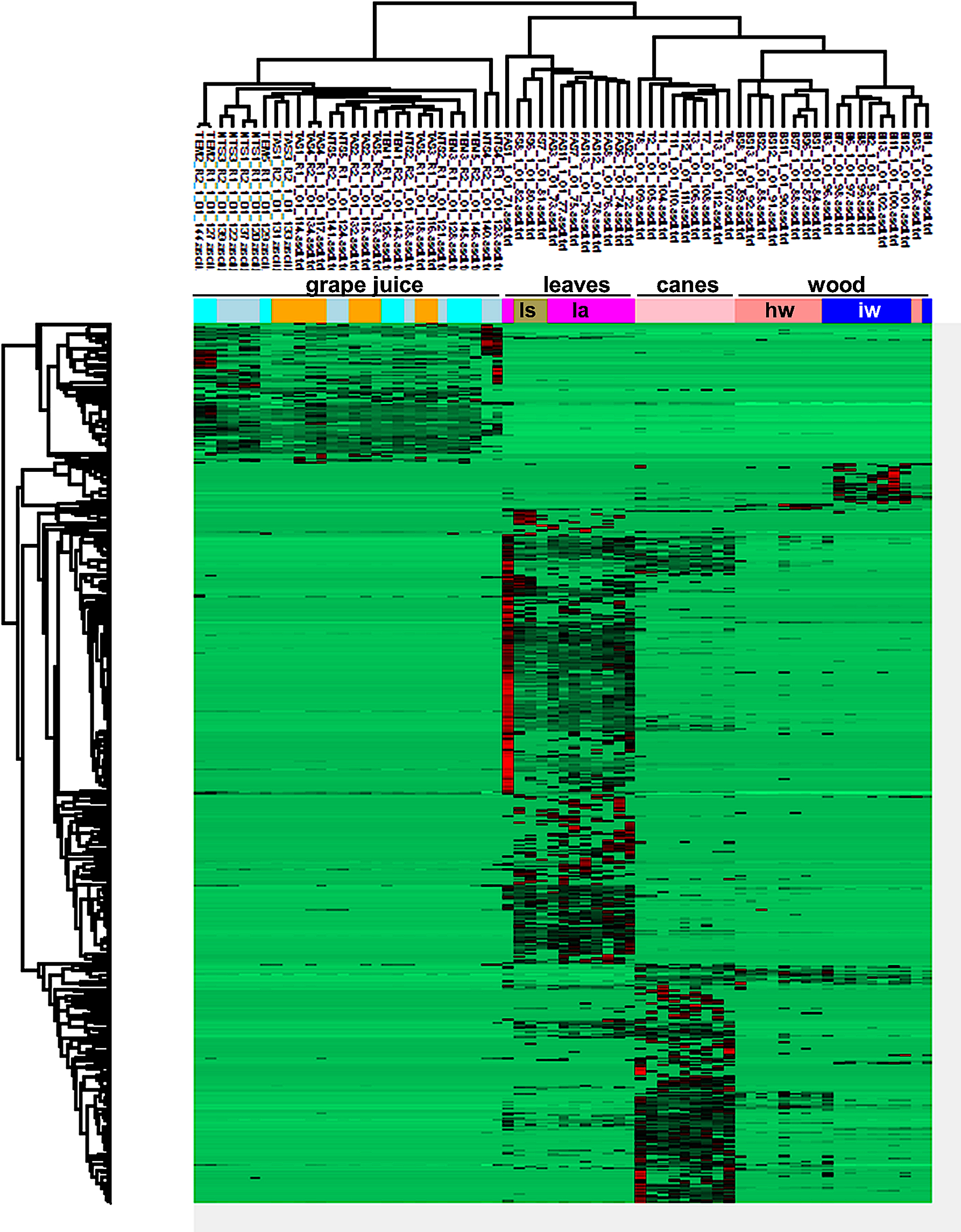

Supplement: Supplementary file 1 [file jof-08-00151-s001.zip › Figure S3.jpg]

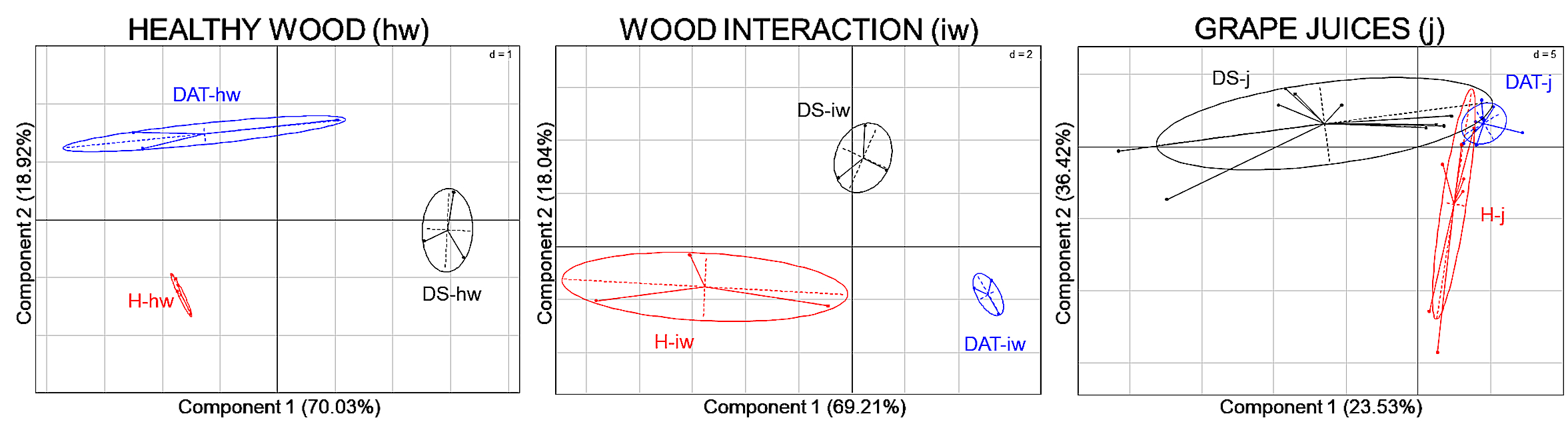

Supplement: Supplementary file 1 [file jof-08-00151-s001.zip › Figure S4.jpg]

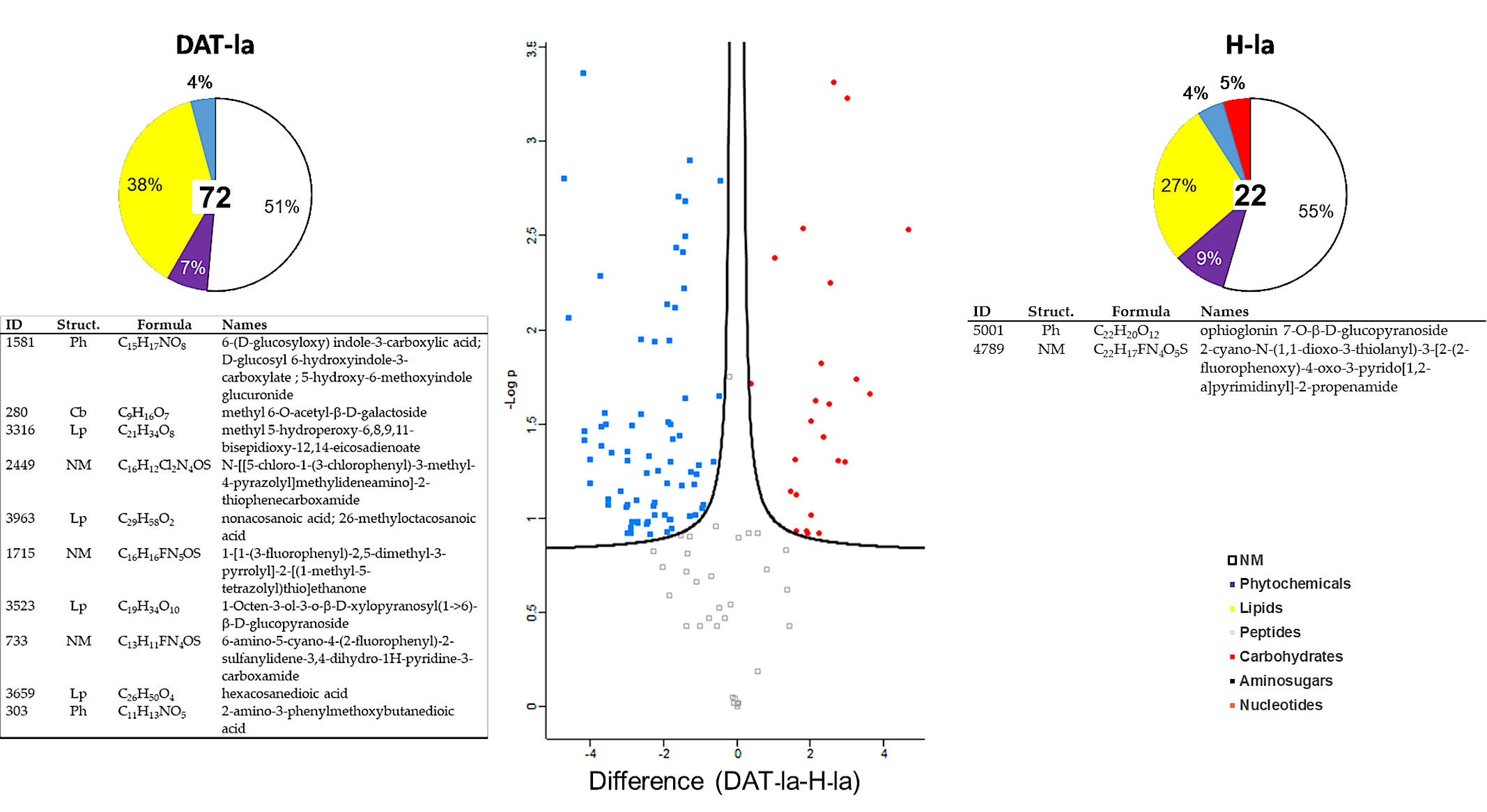

Supplement: Supplementary file 1 [file jof-08-00151-s001.zip › Figure S5.jpg]
